# The androgen receptor expression and its activity have different relationships with prognosis in hepatocellular carcinoma

**DOI:** 10.1038/s41598-020-79177-2

**Published:** 2020-12-16

**Authors:** S. Acosta-Lopez, D. Diaz-Bethencourt, T. Concepción-Massip, M. C. Martin-Fernandez de Basoa, A. Plata-Bello, A. Gonzalez-Rodriguez, F. Perez-Hernandez, J. Plata-Bello

**Affiliations:** 1 Liver Unit, Hospital Universitario Nuestra Señora de Candelaria, 38010 La Laguna, S/C de Tenerife Spain; 2 Hormone Section of the Biochemical Laboratory, Hospital Universitario Nuestra Señora de Candelaria, 38010 La Laguna, S/C de Tenerife Spain; 3 Department of Urology and Androdology, Hospital Universitario de Canarias, 38320 La Laguna, S/C de Tenerife Spain; 4 Department of Neuroscience, Hospital Universitario de Canarias, Calle Ofra s/n La Cuesta, 38320 La Laguna, S/C de Tenerife Spain

**Keywords:** Hepatocellular carcinoma, Molecular medicine

## Abstract

The role of the Androgen Receptor (AR) expression and its activity in the prognosis of hepatocellular carcinoma (HCC) remains inconclusive. The aim of this study is to analyze the role of the AR expression and its activity as prognostic biomarkers in HCC. Three-hundred and thirty-seven patients from The Cancer Genome Atlas (TCGA) (107 females; 59.42 years [SD = 13.0]) were included. To infer AR activity, the expression-profile of previously validated androgen responsive genes (ARGs) was included. AR activity was shown by the AR-Score-21 (21 ARGs) and AR-Score-13 (13 ARGs) that were computed based on the expression of the selected ARGs. Those ARGs whose expression was significantly different between histological grades were used for computing two new AR-Scores. HCC patients with higher AR expression showed a higher median overall survival (OS). AR-Score 21 and AR-Score-13 did not show any association with prognosis. Six of the 21 ARGs of the AR-Score-21 and 7 of the 13 ARGs of the AR-Score-13 showed a significant different expression profile among histological grades. Based on these differences, another two AR-Scores were computed (AR-Score-6 and AR-Score-7). They showed the relative increase of upregulated to downregulated ARGs in high-grade HCC. Higher AR activity inferred by these AR-Scores was associated with worse outcomes. The expression of AR is associated with a better prognosis in HCC. However, the activity of the AR seems to be qualitatively different among histological grades. The AR activity inferred by the shifted ARGs is associated with a worse prognosis in HCC patients.

## Introduction

Hepatocellular carcinoma (HCC) is the most common primary tumor of the liver, with 854,000 new cases/year and it is the second cause of cancer related death around the world (810,000 deaths/year)^1,2^. HCC has a male predominance, with more than double the incidence in men than in women^3,4^. This male dominance has also been reported for other cancers like gastric cancer^5^, bladder cancer^6^ or primary brain tumors^7^. The rationale of this male dominance in these cancers is not completely understood, but it may be associated with sexual hormones or their receptors^8,9^. This association has not completely confirmed unlike other tumors (e.g. prostate cancer or breast cancer) where the role of sexual hormones and their receptors have been studied in depth^10,11^.

The androgen receptor (AR) is a transcription factor that, once activated (preferentially by testosterone or dihydrotestosterone), binds to the DNA in specific regions called androgen response elements (AREs)^12^. The AR regulates the expression of specific target genes, also known as androgen-responsive genes (ARGs) which may or may not have one or more AREs^13^. Many ARGs have been identified and experimentally validated, mainly in prostate cancer cell lines^13,14^. The expression signatures of some of these ARGs have been used to calculate a score that putatively reflects the AR activity and can be used to identify different genomic prostate cancer subtypes^15^. Thus, the use of the ARGs expression profile to infer AR activity seems to be an adequate tool to study the role of AR activity in cancer.

However, ARGs may alter their expression profile depending on the microenvironment. In this respect, Pomerantz et al. showed that the AR binds to specific AREs in tumoral cells and normal prostate cells, in different ways^16^. In other words, the ARGs expression profile seems to be different in tumoral and in normal tissues^16,17^. Furthermore, the ARGs expression also seems to be related with the period when the AR is exposed to androgens^18^. Therefore, AR activity and its associated up- or downregulated ARGs seem to vary among different microenvironmental and cellular conditions.

The role of AR and its activity in HCC has not been fully studied. The AR is present in HCC, but also in normal surrounding parenchyma and normal liver^19^. Generally speaking, it has been demonstrated that AR expression is variable, but a higher expression in tumoral tissue than in normal surrounding tissue has been described^20–22^. Furthermore, the AR has been associated with hepatocarcinogenesis^23–25^. However, published literature about the relationship of the AR with HCC pathogenesis and evolution is controversial. On the one hand, a relationship between protein and mRNA AR expression with a better prognosis has been reported^26^. On the other hand, other authors have described a positive correlation between the AR expression at nucleus with more advanced tumoral stages and, consequently, with a worse prognosis^20^. In this regard, the recurrence rate of HCC patients was higher in patients with AR expression in the tumoral surrounding tissue and the survival rate was better in AR negative patients^8^. Consequently, more research about the role of the AR and, mostly, about the role of the AR activity is required. This is especially important if one bears in mind that there are various specific therapeutic approaches to block the AR pathways that could be useful for HCC management.

Therefore, the aim of the present work is to study the role of the AR expression and its putative activity as a prognostic biomarker in HCC in a cohort of patients from The Cancer Genome Atlas (TCGA) project.

## Methods

### Patients

Three hundred and sixty-three (119 females; mean age 59.79 years [SD = 13.05]) were included from the TCGA database (from a whole cohort of 377 patients). All patients presented a confirmed HCC (fibrolamellar carcinoma patients were previously excluded [n = 3]). Only patients whose tumor samples have at least RNAseq data available were included (n = 363). Furthermore, patients whose pathologic stage was not registered were also excluded (n = 25). Thus, a final cohort of three hundred and thirty-seven patients were analyzed. Sample features are shown in Supplementary Table S1.

### TCGA data extraction

Data from TCGA was downloaded from Firebrowse (http://firebrowse.org/) (TCGA data version 2016_01-28). As mentioned above, only patients with RNAseq data available were included in the study. Apart from RNAseq data, clinical, mutational, copy number variations (CNVs) and reversed phase protein array (RPPA) data from the selected patients were also downloaded and included in a new database. It should be stated that all clinical and molecular data were not available for all patients. Details of this data generation are described elsewhere^27^. The study has been approved by the Hospital Universitario de Canarias’ ethics committee. Informed consent was obtained from all subjects by TCGA researchers. All experiments were performed in accordance with relevant guidelines and regulations.

### AR RNAseq data

AR-RNA expression data were extracted as explained above and did not have a normal distribution (Kolmogorov–Smirnov; p < 0.05), thus a dichotomization was performed using the median (111.24 RPKM). This variable was used to compare those patients with low and high AR-RNA expression with one another. Furthermore, a survival analysis was performed to determine differences in median overall survival (OS) and median progression free survival (PFS) between the two AR-RNA expression groups.

### AR RPPA data

AR-protein expression data were extracted as described above. The determination of AR expression in the RPPA was performed using a specific antibody (ab52615, Abcam). RPPA data were already normalized and the AR expression had a normal distribution (Kolmogorov–Smirnov; p = 0.552). Furthermore, AR-protein expression was also dichotomized (using the median as cutoff; p50 = − 0.0204) and this variable was used for clinical and molecular data comparisons, as well as Kaplan–Meier curves and Log-Rank test analysis.

### AR putative activity: the AR-Score

Apart from AR expression data (at protein and RNA levels), other gene expression profiles were also extracted. All of these genes have been previously validated as androgen response transcriptional targets (ARGs)^13,14^. They were used to infer the activity of the AR. As other studies did, the activation of the AR pathway was indicated by the AR-Score^15,26^. These previous publications computed the AR-Score based on the 21 ARGs expression reported by Hieronymus et al. This AR-Score was also computed in the present work, but another AR-Score, based on the expression of 13 ARGs expression reported by Bolton et al. was also computed. To distinguish one score from the other, the first one has been called the AR-Score-21 and the second one the AR-Score-13 (the number represents the number of genes included in each AR-Score).

As described in other works^15^, a Z-score was computed for the expression of each gene in each sample by subtracting the pooled mean from the RNAseq expression values and dividing the result by the pooled standard deviation. Both AR-Scores for each sample were computed as the sum of the Z-scores of each ARGs signature. None of the AR-Scores presented a normal distribution (Kolmogorov–Smirnov; AR-Score-21, p = 0.023; AR-Score-13, p = 0.004). Both AR-Scores were also dichotomized (using the median) and this variable was used to compare groups of a low (< = p50) or high (> p50) AR-Score for Kaplan–Meier curves and Log-Rank test analysis.

Finally, regarding the fact that the ARGs expressions is different in normal and in tumoral tissue^17^ (i.e. that the AR shows more sensitivity to certain targets for either normal or tumoral tissue), one can hypothesize that this variation may also be present in different tumoral histological grades. To confirm this hypothesis, the expression of the genes included in each AR-Score was compared between histological grades. Those genes whose expression was significantly different between histological grades were used for computing two new AR-Scores (one for the AR-Score-21 significantly different expressed genes and another for those from the AR-Score-13).

In these two new AR-Scores, the relative increase of genes that are upregulated to those genes that are downregulated in higher histological grades (HG) was considered. They have been computed as follows:

$$ \# \;{\text{AR-Score-new}} = {{\left( {\sum \;\left[ {{\text{upregulated}}\;{\text{genes}}\;{\text{expression}}\;{\text{in}}\;{\text{high}}\;{\text{HG}}} \right]} \right)} \mathord{\left/ {\vphantom {{\left( {\sum \;\left[ {{\text{upregulated}}\;{\text{genes}}\;{\text{expression}}\;{\text{in}}\;{\text{high}}\;{\text{HG}}} \right]} \right)} {\left( {\sum \;\left[ {{\text{downregulated}}\;{\text{genes}}\;{\text{expression}}\;{\text{in}}\;{\text{high}}\;{\text{HG}}} \right]} \right)}}} \right. \kern-\nulldelimiterspace} {\left( {\sum \;\left[ {{\text{downregulated}}\;{\text{genes}}\;{\text{expression}}\;{\text{in}}\;{\text{high}}\;{\text{HG}}} \right]} \right)}} $$

The two new AR-Scores were also dichotomized using the median as cutoff and they were used for survival analysis in the same way as the other AR-Scores.

### DNA copy-number variation and DNA mutations

Copy number variation and mutation annotation files were downloaded as described above and they have been analyzed as described in previous reports^27^. After excluding those patients whose RNAseq data was not available, the ten-most-common cancer related genes that show copy number alteration (CNA) and/or mutation were studied. Those genes (Supplementary Tables S2 and S3) were identified from the whole TCGA hepatocellular carcinoma cohort whose analysis is shown in cBioPortal (https://www.cbioportal.org/). Comparative analysis of the distribution of these genetic events between the two expression groups of AR (RNA and protein) and AR-Scores was performed.

### Statistical analysis

As previously stated, the median was used to generate two comparable groups for each data to study the differences between low and high AR and AR-Scores expression. Nonparametric statistical tests were used (Mann–Whitney U or Kruskal Wallis for continuous variables and Chi-Square/Fisher exact test for discrete variables). Statistical significance was considered when the p-value < 0.05. However, bearing in mind the high number of comparisons during molecular analysis, a corrected p value was used for these variables, using the False Discovery Rate (FDR) method. Differences were considered statistically significant when FDR < 0.1.

Kaplan–Meier curves and the Log-Rank test were used to study the differences in overall survival (OS) and progression free survival (PFS) between different AR (RNA and protein) and AR-Scores expression groups. The statistical significance for survival analysis was considered when the p-value < 0.05.

Finally, correlation analysis between AR-Score and AR expression (at RNA and protein levels) was performed using the Spearman Rho test (p-value < 0.05).

## Results

### AR expression in hepatocellular carcinoma

RNAseq data showed a mean AR expression of 257.9 RPKM (Reads Per Kilobase Million) (SD = 381.0) and Reversed Phase Protein Analysis (RPPA) data showed a mean AR abundance of − 0.01 (SD = 0.31) in the probes of HCC. As expected, a significant positive correlation between AR at the protein and mRNA levels was found (Spearman’s Rho; Correlation Coefficient = 0.305; p = 0.0001).

Analysis of copy number variations (CNVs) showed that no patient presents an amplification or deletion of the AR gene. AR mutations were identified in 3 patients (0.9%). Two of these were missense mutations and the other was a truncating mutation. In any case, none of them were considered driver mutations.

### Comparison between AR-RNA expression groups

A comparative analysis between patients with low (< = p50) and high AR-RNA expression (> p50) was performed using the median of AR-RNA expression (p50 = 111.24; n = 337). Patients in the high AR-RNA group were older than those in the low AR-RNA group (61.6 vs. 57.3 years; p = 0.001). Gender distribution also shows differences between groups (higher proportion of females in low AR-RNA group [37.8% vs. 25.6%]; p = 0.019). Significant differences were also identified in the histologic grade distribution (p = 0.002) and vascular invasion (p = 0.014). Low AR-RNA patients presented higher histologic grades (G3 and G4) and a higher proportion of cases with micro- (31.7%) and macrovascular invasion (9.2%) (Supplementary Table S4). According to these histological findings, patients in the low AR-RNA group presented more molecular changes, with a higher mean of fraction of genome altered (0.3 vs. 0.25; p = 0.0002) and mean aneuploidy score (12.8 vs. 8.3; p < 0.0001) (Supplementary Table S4).

Analysis of CNVs in the top-10 HCC cancer-related genes with focal CNVs showed a larger number of amplifications in MYC (4q12) in low AR-RNA patients (FDR = 0.07) (Supplementary Table S5). On the other hand, a higher proportion of driver mutations in the CTNNB1 gene were identified in the high AR-RNA group (FDR = 0.03) (Supplementary Table S6).

**Figure 1:**
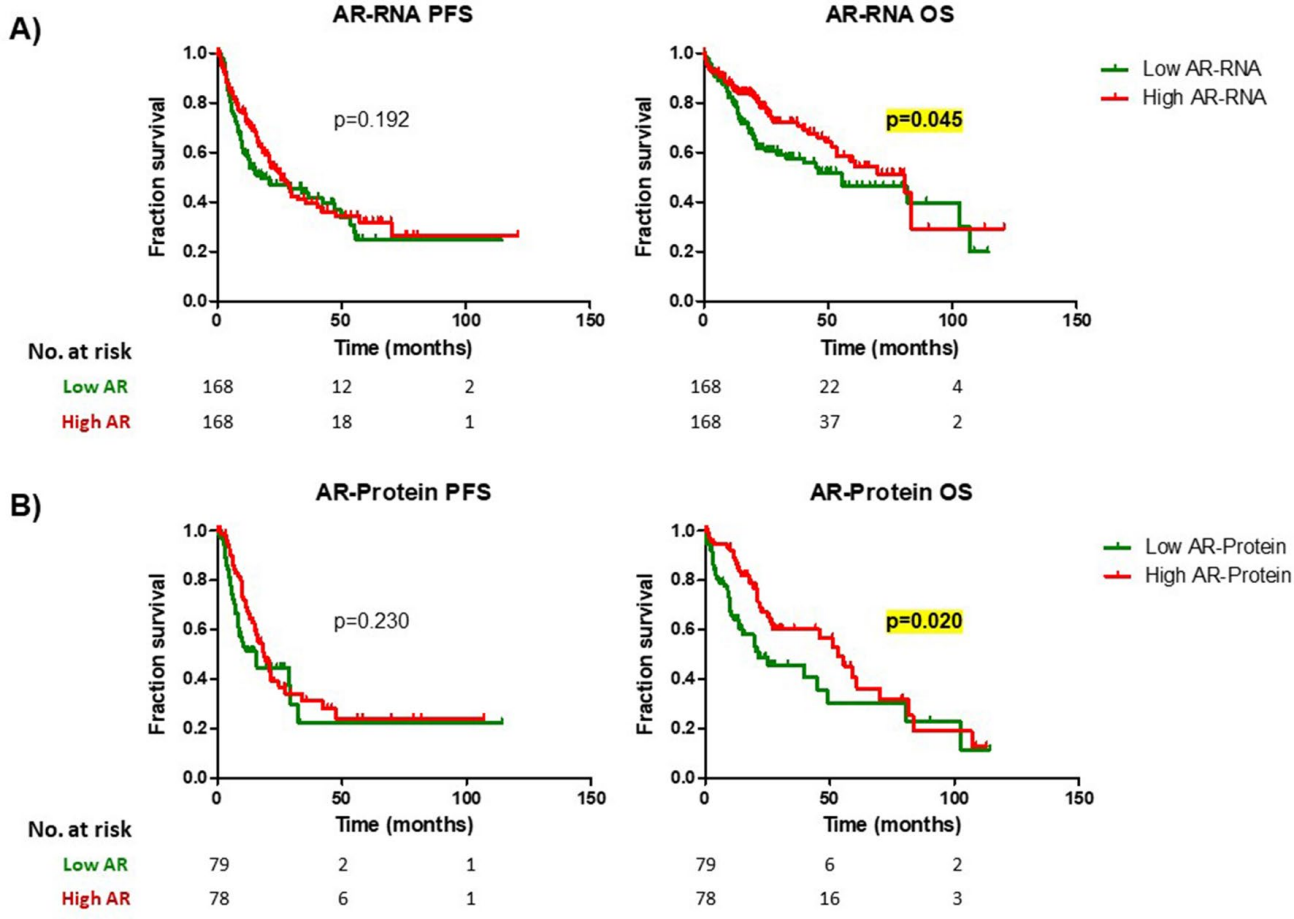
Survival analysis (progression free survival [PFS] and overall survival [OS]) in different androgen receptor expression groups at the level of mRNA (**A**) and protein (**B**).

The survival analysis showed that patients in the low AR-RNA group presented a significant shorter OS (55.4 months, 95% C.I. [26.9–83.8]) than patients in the high AR-RNA group (80.7 months, 95% C.I. [48.7–91.4]) (Log-Rank test; p = 0.045) (Fig. 1A; Supplementary Table S4). No differences between low and high AR-RNA groups were found in median PFS (17.6 vs 25.5 months; p = 0.192) (Fig. 1A; Supplementary Table S4).

### Comparison between AR-protein expression groups

As performed with AR-RNA, the median of AR-protein expression (p50 = − 0.20; n = 157) was used to generate two groups of patients (i.e. low and high AR-protein expression groups). Clinical and molecular variables were compared between these two groups. These comparisons did not show any statistically significant difference (Supplementary Table S7). In the same vein, no differences in the analysis of CNVs (Supplementary Table S8) and mutational signature (Supplementary Table S9) were identified.

Regarding the survival analysis, the median OS in patients with high AR-protein was 53.3 months (95% C.I. 40.6–66.0) while the median OS in patients with low AR-protein was 21.3 (95% C.I. [3.8–38.9]) (Fig. 1B; Supplementary Table S7). This difference was statistically significant (Log-Rank test, p = 0.020). No significant differences in median PFS were identified between low and high AR-protein expression (15.6 vs. 18.6 months; p = 0.230) (Fig. 1B; Supplementary Table S7).

### AR activity measured by AR-Score-21 and AR-Score-13

**Figure 2:**
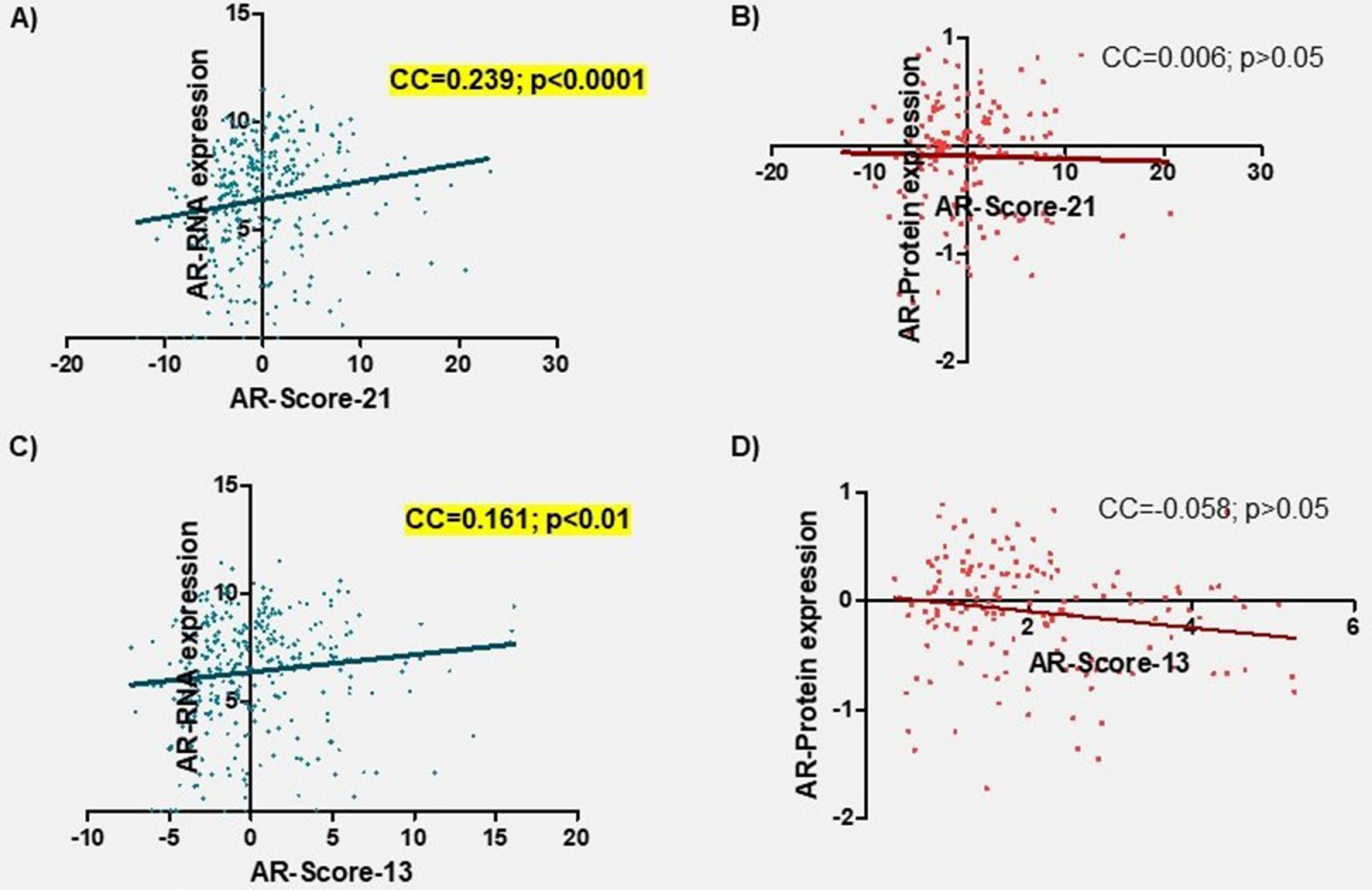
Correlation analysis between androgen receptor expression at RNA and protein levels and the AR-Score-21 (**A**,**B**) and AR-Score-13 (**C**,**D**). Spearman Rho test was used to calculate the correlation coefficients and p-values. All variables have been transformed using log2. *CC* correlation coefficient.

As explained in “Methods”, the AR activity was estimated using two scores that were computed by the sum of previously validated AR targets (see “Methods” and Supplementary Table S22). The mean of the AR-Score-21 was − 0.0027 (SD = 5.4). The AR-Score-21 was positively correlated with AR-RNA expression (CC = 0.239; p = 0.000009) (Fig. 2A). On the other hand, the mean of the AR-Score-13 was − 0.0003 (SD = 3.8). AR-Score-13 also showed a positive correlation with AR-RNA expression (CC = 0.161; p = 0.003) (Fig. 2C). A moderate correlation between both AR-Scores was also found (CC = 0.454; p < 0.00001). None of the AR-Scores presented a correlation with AR-protein expression (Spearman Rho; p > 0.05) (Fig. 2B,D).

As performed with AR-RNA and AR-protein, two groups of AR-Score values (2 for each AR-Score) were generated using the median as cutoff (p50 AR-Score-21 = − 0.72 and p50 AR-Score-13 = − 0.72). Comparisons between groups were made.

Firstly, regarding the comparison between low and high AR-Score-21 groups, a higher fraction of genome altered (p = 0.001) and aneuploidy score (p = 0.001) were found in low AR-Score-21 patients (Supplementary Table S10). CNVs and mutation signature analysis did not show any significant differences between AR-Score-21 expression groups (Supplementary Tables S11 and S12).

**Figure 3:**
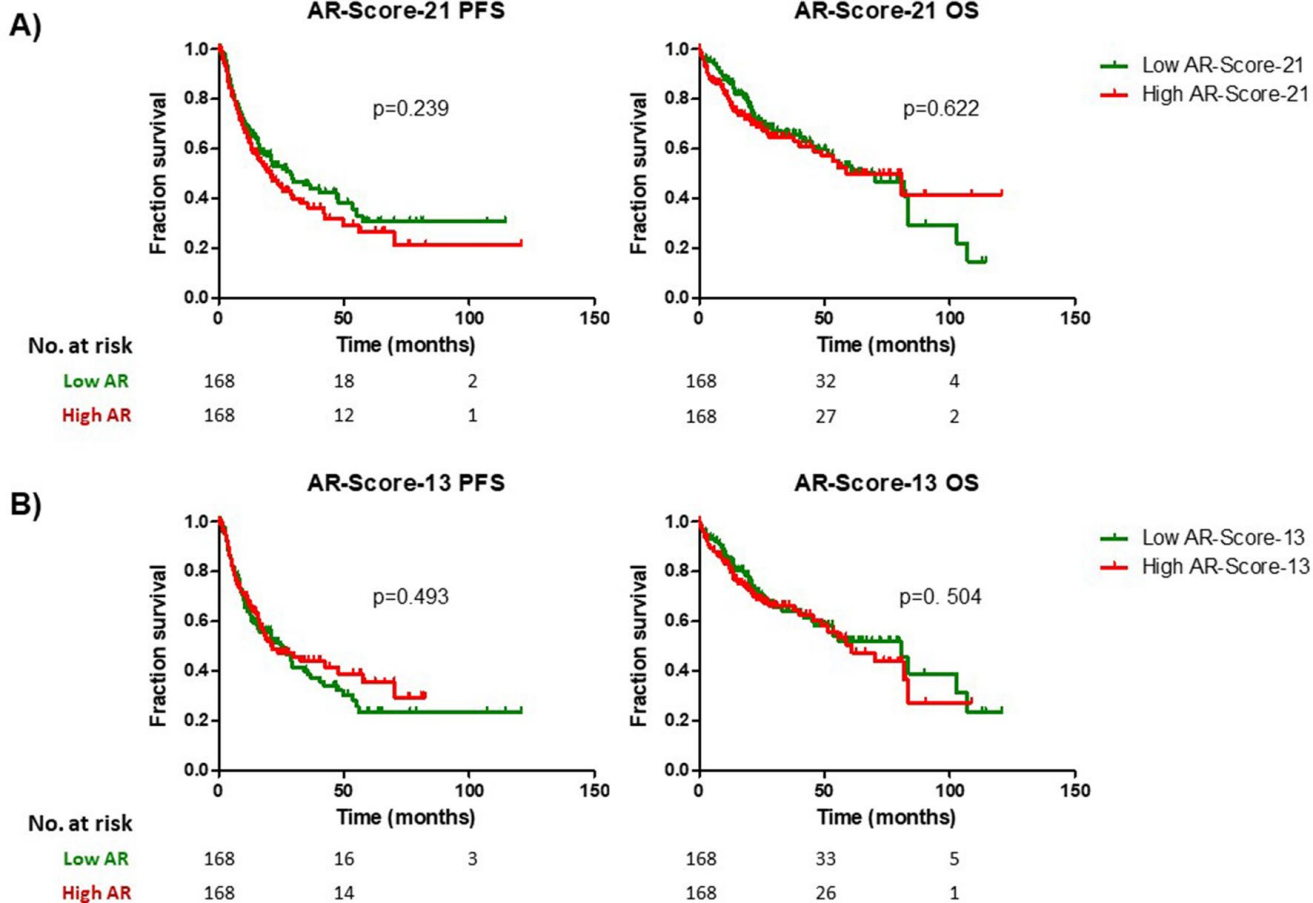
Survival analysis (progression free survival [PFS] and overall survival [OS]) in different AR-Score-21 (**A**) and AR-Score-13 groups (**B**).

In the survival analysis, the median PFS in the low AR-Score-21 group was 28.8 months (95% C.I. [16.3–41.2]) and 20.9 (95% C.I. [13.9–27.9]) in the high AR-Score-21 group, without statistical significance (Log-Rank; p = 0.239) (Fig. 3A; Supplementary Table S10). The median OS in the low AR-Score-21 group was 70.1 months (95% C.I. [44.7–95.4]) and 58.9 months (95% C.I. [34.5–83.2]) in high AR-Score-21 was. This different did not reach statistical significance either (Log-Rank; p = 0.622) (Fig. 3A; Supplementary Table S10).

Secondly, differences between low and high AR-Score-13 were identified in the histological grade distribution (higher rates of grade 3 in low AR-Score-13 group; p = 0.05); and more genetic alterations were present in the low AR-Score-13 group (higher means of mutation count [p = 0.027], fraction of genome altered [p = 0.04] and aneuploidy score [p = 0.002]) (Supplementary Table S13). CNVs analysis showed higher rates of amplification of CKS1B and RIT1 (FDR = 0.022 and FDR = 0.028) in the high AR-Score-13 group (Supplementary Table S14). No significant differences in mutation signature analysis were found between the AR-Score-21 expression groups (Supplementary Table S15).

In the survival analysis, the median PFS in the low AR-Score-13 group was 25.5 months (95% C.I. [15.9–35.1]) and 21.0 months (95% C.I. [9.3–32.7]) in high AR-Score-13 group, without statistical significance (Log-Rank; p = 0.493) (Fig. 3B; Supplementary Table S13). The median OS in low AR-Score-13 group was 80.7 months (95% C.I. [53.8–107.7]) and 60.9 months (95% C.I. [40.8–80.9]) in high AR-Score-13. This difference did not reach statistical significance either (Log-Rank; p = 0.504) (Fig. 3B; Supplementary Table S13).

### AR-Score-21 and AR-Score-13 genes switch their expression with different tumoral histological grades: the AR-Score-6 and the AR-Score-7

As mentioned above, ARGs expression can vary between normal and tumoral tissue and this variation may also be present in different tumoral histological grades. This variation may have prognostic implications. Therefore, an analysis was conducted of the changes in the genetic expression profile between histological grades in each AR-Score. New AR-Scores were computed and tested in survival analysis with this information.

**Figure 4:**
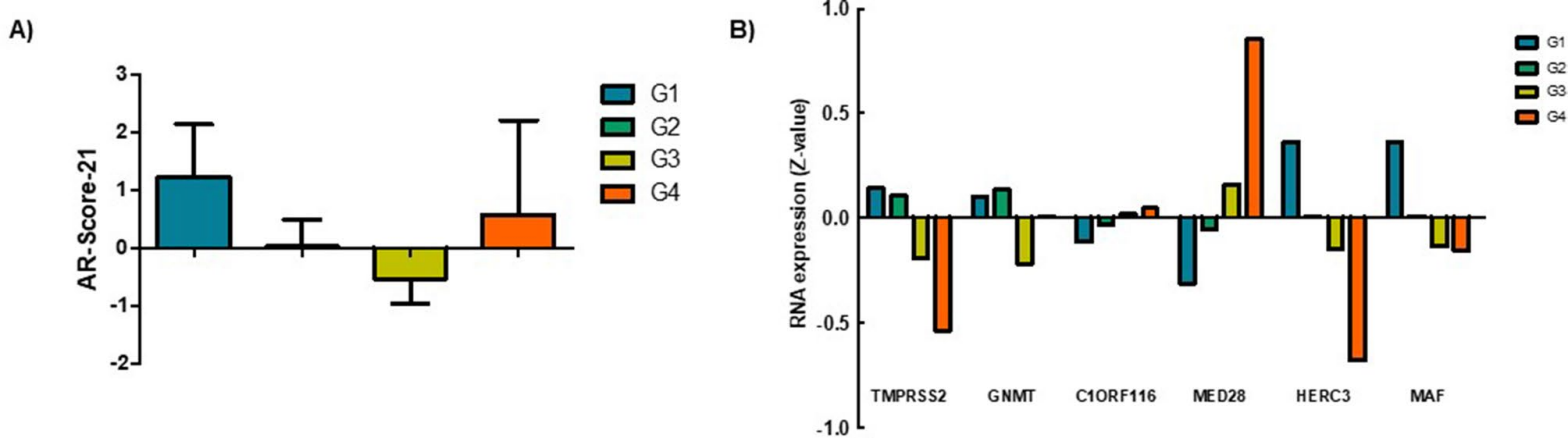
(**A**) AR-Score-21 in each histological grade. No statistical differences were identified between groups. Bars represent the mean of AR-Score-21 values, and whiskers the standard deviation in each histological grade. (**B**) Expression profile of the genes that show differences among histological grades. Bars represet the mean normalized expression of the indicated genes in each histological grade.

On the one hand, no difference in the mean AR-Score-21 was identified among histological grades (Kruskal–Wallis; p = 0.609) (Fig. 4A), but a different profile of genetic expression in 6 of the its 21 genes was identified (Fig. 4B). C1ORF116 (p = 0.039) and MED28 (p = 0.013) showed more expression in high histological grades of HCC samples. On the other hand, TMPRSS2 (p = 0.009), GNMT (p = 0.030), HERC3 (p = 0.003) and MAF (p = 0.023) showed greater expression in low histological grades HCC samples. Based on these differences, another AR-Score was computed (called AR-Score-6). This score shows the relative increase of upregulated genes in high grade HCC in relation to those which are downregulated (all of these genes are included in the AR-Score-21). This score was calculated as follows:

$$ \# \;{\text{AR-Score-}}6 = \left( {{\text{C}}1{\text{ORF}}116 + {\text{MED}}28} \right)/\left( {{\text{TMPRSS}}2 + {\text{GNMT}} + {\text{HERC}}3 + {\text{MAF}}} \right) $$

Gene symbols represent the normalized (Z) RNA expression of each gene.

The AR-Score-6 did not have a normal distribution (Kolmogorov–Smirnov; p < 0.05), thus a dichotomization of the variable using the median (p50 = 0.12) as cutoff was performed. Patients with low AR-Score-6 were older than high AR-Score-6 patients (61.1 vs. 57.8 years; p = 0.033) (Supplementary Table S16). A different distribution in pathologic stages was found with higher rates of stages II and III in the high AR-Score-6 group (p = 0.021) (Supplementary Table S16). Regarding molecular variables, a higher mean of fraction genome altered and aneuploidy score were found in high AR-Score-6 patients (p = 0.0002 and p = 0.00004) (Supplementary Table S16). CNVs and mutation signature analysis did not show any significant difference between AR-Score-6 expression groups (Supplementary Tables S17 and S18).

In the survival analysis, patients in the high AR-Score-6 group had a median OS of 58.9 months (C.I. 95% [42.7–75.1]) while those patients in the low AR-Score-6 group presented a median OS of 83.2 months (C.I. 95% [43.2–123.3]) (supplementary Table S16, Fig. 6A). This difference did not reach statistical significance (Log-Rank test; p = 0.153). The high AR-Score-6 group presented a significantly worse PFS than the low AR-Score-6 group (16.5 vs. 32.5 months; p = 0.005) (Supplementary Table 16, Fig. 6A).

**Figure 5:**
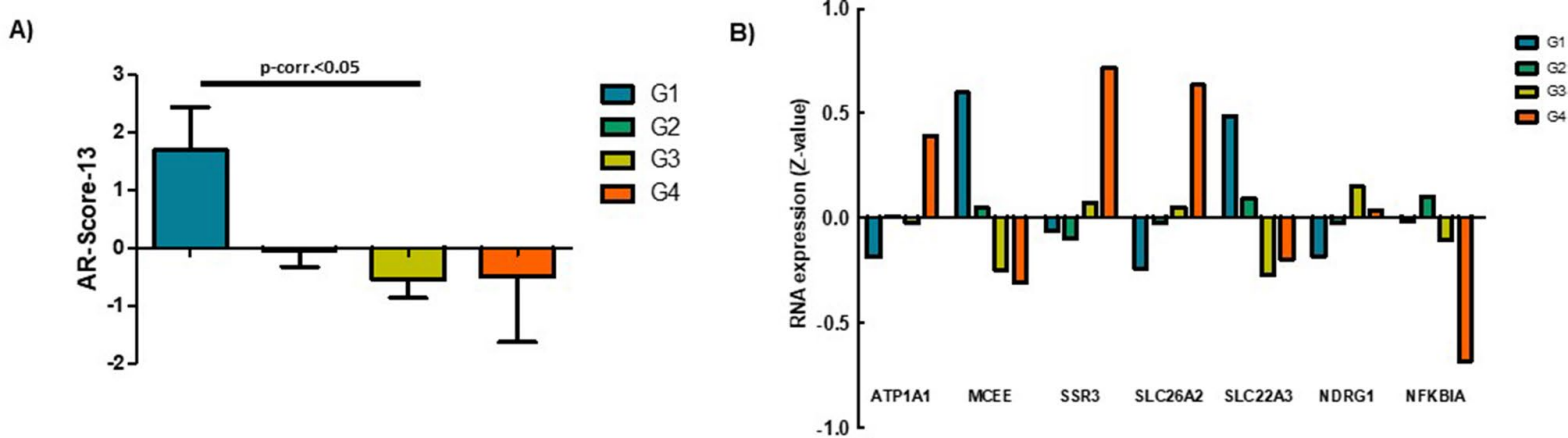
(**A**) AR-Score-13 in each histological grade. Significant differences between Grade I and Grade III were identified. Bars represent the mean of AR-Score-13 values, and whiskers the standard deviation in each histological grade. (**B**) Expression profile of the genes that show differences among histological grades. Bars represet the mean normalized expression of the indicated genes in each histological grade.

On the other hand, a difference in mean AR-Score-13 was found among histological grades (Kruskal–Wallis; p = 0.032). The main difference was identified between grades 1 and 3 (Dunn’s multiple comparison test; p < 0.05) (Fig. 5A). Interestingly, a different profile of genetic expression in 7 of the 13 genes was identified (Fig. 5B). An increased expression with histologic grade was found for ATP1A1 (p = 0.0002), SSR3 (p = 0.043), SLC26A2 (p = 0.005) and NDRG1 (p = 0.014). On the other hand, a decreased expression with histologic grade was shown for MCEE (p = 0.00007), SLC22A3 (p = 0.0001) and NFKBIA (p = 0.0002). Based on these differences, a new AR-Score was computed (called AR-Score-7). This new score shows the relative increase of upregulated genes in higher histological grades in relation to those which are downregulated (all of these genes are included in the AR-Score-13). This new AR-score was calculated as follows:

$$ \# \;{\text{AR-Score-7}} = \left( {{\text{ATP1A1}} + {\text{SSR3}} + {\text{SLC26A2}} + {\text{NDRG1}}} \right)/\left( {{\text{MCEE}} + {\text{SLC22A3}} + {\text{NFKBIA}}} \right) $$

Gene symbols represent the normalized (Z) RNA expression of each gene.

The AR-Score-7 data did not have a normal distribution (Kolmogorov–Smirnov; p < 0.05), thus a dichotomization of the variable using the median (p50 = 2.10) as cutoff was performed. Patients in the high AR-Score-7 group were younger (58.0 vs. 61.1 years; p = 0.016) and with a larger female representation (37.2% vs. 26.1%; p = 0.035) than patients with low AR-Score-7 (Supplementary Table S19). It is worth mentioning that the high AR-Score-7 group was associated with higher pathologic stages (32.5% in stage III) than the low AR-Score-7 group (17.3% in stage III) (Chi-Square; p = 0.005) (Supplementary Table S19). Furthermore, a higher incidence of driver mutations in TP53 was identified in the high AR-Score-7 group (38.7% vs. 22.0%; FDR = 0.03) (Supplementary Table 21). On the contrary, a higher incidence of driver mutations in the CTNNB1 gene was found in the low AR-Score-7 group (34.1% vs. 17.9%; FDR = 0.04) (Supplementary Table S21). CNVs analysis did not show any significant difference between AR-Score-7 expression groups (Supplementary Table S20).

**Figure 6:**
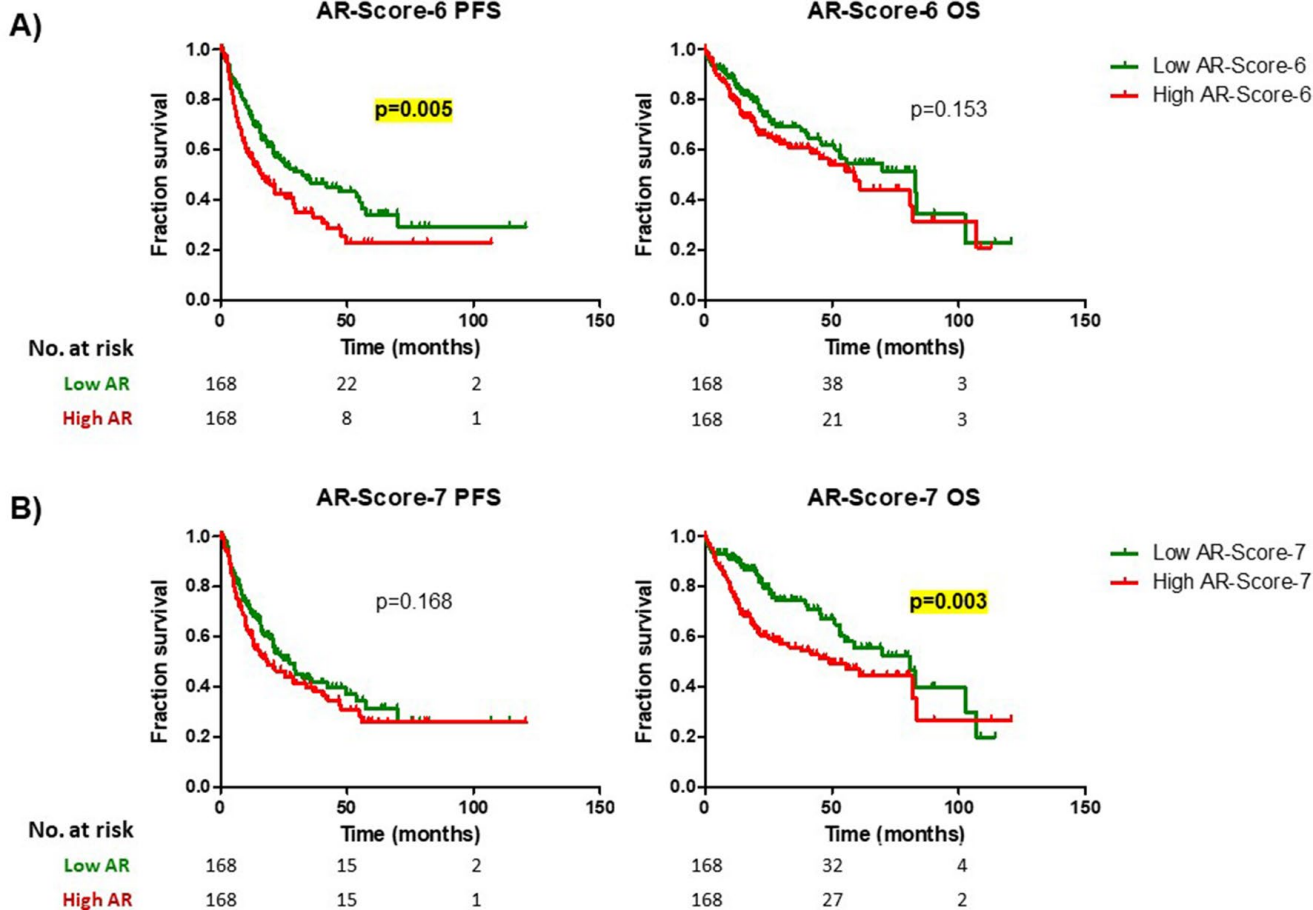
Survival analysis (progression free survival [PFS] and overall survival [OS]) in different AR-Score-6 (**A**) and AR-Score-7 groups (**B**).

In the survival analysis, the high AR-Score-7 patients had a median OS of 49.0 months (C.I. 95% [25.1–72.9]) while those patients in the low AR-Score-7 group had a median OS of 80.7 months (C.I. 95% [56.0–105.5]) (Supplementary Table S19; Fig. 6B). This difference was statistically significant (Log-Rank test; p = 0.003). The median PFS was not different between the low and high AR-Score-7 groups (27.2 vs. 18.4 months; p = 0.168) (Supplementary Table S19; Fig. 6B).

**Figure 7:**
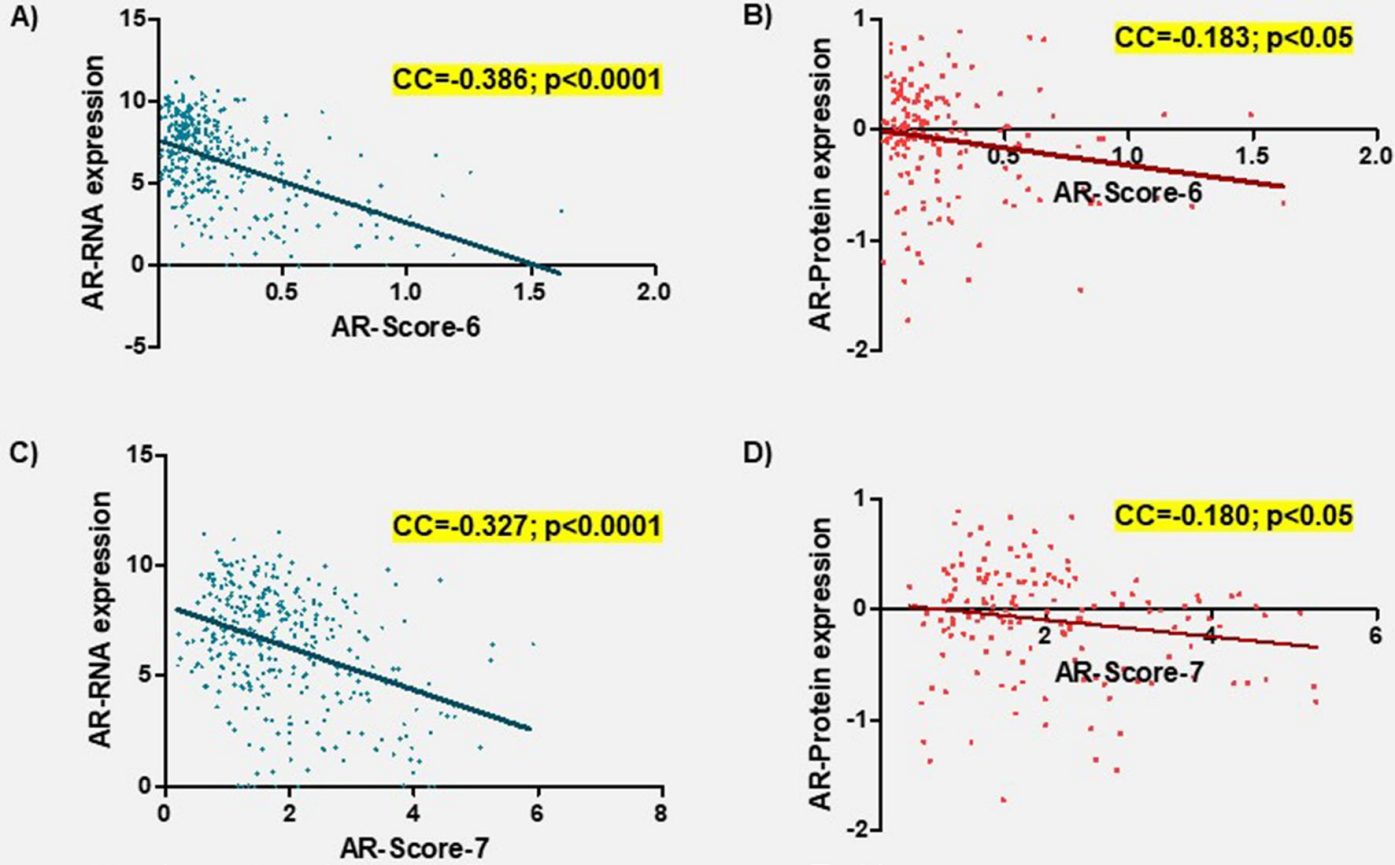
Correlation analysis between androgen receptor expression at RNA and protein levels and the AR-Score-6 (**A**,**B**) and AR-Score-7 (**C**,**D**). Spearman Rho test was used to calculate the correlation coefficients and p-values. All variables have been transformed using log2. *CC* correlation coefficient.

Finally, a correlation analysis showed a weak negative correlation between both new AR-Scores (AR-Score-6 and AR-Score-7) with AR-RNA expression (CC = − 0.386 and CC = − 0.327; p < 0.0001) (Fig. 7A,C) and AR-protein expression (CC = − 0.183 and CC = − 0.180; p < 0.05) (Fig. 7B,D).

### Correlation analysis between AR expression and AR-Scores in different tumor stages

A correlation analysis between AR expression and AR-Scores in different tumor stages (American Joint Committee on Cancer staging system) was performed. In stage I patients (167), a significant weak positive relationship between AR at RNA level and AR-Score-21 and AR-Score-13 was identified (CC = 0.253 and CC = 0.201, respectively; p < 0.01). Furthermore, in this group of patients, a weak negative correlation between AR at RNA level and AR-Score-6 and AR-Score-7 was identified (CC = − 0.252 and CC = − 0.177, respectively; p < 0.05). Regarding patients with stage II (86), a significant weak positive relationship between AR at RNA level and AR-Score-21 and AR-Score-13 was identified (CC = 0.390 and CC = 0.229, respectively; p < 0.05). In this group of patients, a weak negative correlation between AR at RNA level and AR-Score-6 and AR-Score-7 was also identified (CC = − 0.471 and CC = − 0.287, respectively; p < 0.01). Finally, in stage III patients (84) no relationship between AR at RNA level and AR-Score-21 and AR-Score-13 was identified. However, a moderate negative correlation between AR at RNA level and AR-Score-6 and AR-Score-7 was identified (CC = − 0.472 and CC = − 0.542, respectively; p < 0.01). No relationship between AR-Scores and AR expression at protein level was identified in any tumoral stage group.

## Discussion

The present study analyzes the expression profile of AR (at mRNA and protein levels) and different ARGs (synthesized in different AR-scores) in HCC and determines their prognostic value. On the one hand, a shift in the ARGs expression profile between low and high histological HCC grades was observed. On the other hand, the activity of the AR was associated with a worse prognosis. However, AR expression seems to be associated with better outcomes. Thus, the activity of the AR and its expression have opposite effects in HCC patients. All these findings will be discussed below.

### Androgen responsive genes shift their expression profile with histological grade

ARGs expression can vary between normal and tumoral tissues. This has previously been demonstrated in prostate cancer. Pomerantz et al. compared prostate cancer and normal prostate tissues and concluded that the AR had specific AREs in each tissue^16^. Sharma et al. reported the existence of specific AREs for each tumoral stage^28^. Furthermore, Wang et al. described a different expression profile when the activation of the AR in prostate cancer was dependent on or independent from androgens^29^.

However, the shift in the ARGs expression profile has not been described in HCC until now. Indeed, the two selected sets of validated ARGs showed a differential expression according to the histological grade (Figs. 4 and 5). Some of these genes have previously been studied in HCC. GNMT (glycine N-methyltransferase) and MAF (MAF bZIP transcription factor) have been reported as tumor suppressor genes in HCC^30,31^ and, therefore, they are both downregulated in higher histological grades (Fig. 4). However, ATP1A1 (ATPase Na + /K + transporting subunit alpha 1) has been associated with proliferation and cellular migration in HCC^32^. As also found in the present work, SSR3 (signal sequence receptor subunit 3) and NDRG1 (N-myc downstream regulated 1) upregulation is associated with a higher histological grade (Fig. 5) and a worse prognosis^33,34^. In brief, a shift in the expression of ARGs has been identified in HCC probes according to the histological grade and of these ARGs, those acting as tumor suppressor genes are upregulated in lower histological grades and their expression decreases in higher histological grades. Conversely, those ARGs with oncogenic functions, are downregulated in lower histological grades and increase their expression while the histological grade progresses.

### Prognostic value of the AR activity measurement

As described above, few studies have analyzed the expression of the AR in HCC and they report contradictory results regarding its relationship with prognosis. Hu et al. described a similar relationship between AR expression and a better prognosis, based on the same TCGA cohort of patients described in the present study^26^. Boix et al. found that HCC patients with higher AR expression presented a lower tumor burden^35^. However, Zhang et al. described a positive correlation between AR level and tumor size and better overall survival in patients with lower AR expression^36^.

Although most of these studies did not specifically measure the activity of the AR, some deductions can be made from their results. For example, Zhang et al. described a worse prognosis of patients with a higher nuclear expression of the AR^20^. The inactive AR is normally located in the cytoplasm and once activated, a translocation to the nucleus occurs^12^. In this regard, the results of Zhang et al. may be more associated with the activation of the AR and not just with the presence of the receptor. In fact, the cohort of patients with high nuclear AR in the work of Zhang et al., presented a more advanced histological stage and a more advanced clinical tumoral stage^20^.

Moreover, Ma et al. reported that the activation of AR in HCC cell lines is associated with higher growth rates and reduced apoptosis^37^. In the same vein, Ao et al. described a higher migration and invasion capacity in vitro related to the activation of the AR^38^.

All these results are compatible with one of the main findings of the present work, where greater AR activity was associated with a worse prognosis. However, it should be noted that a modification of the initial AR-Scores (i.e. AR-Score-21 and AR-Score-13) was made, taking into consideration the shift of the ARGs in higher histological grades as mentioned above. In this respect, the new AR-Scores (i.e. AR-Score 6 and AR-Score 7) seem to reflect the oncogenic pathways activated through the activation of the AR in HCC (see “Methods”).

The relationship between the modified AR-Scores with a worse prognosis showed that the presence of the AR is not enough to associate a worse prognosis to HCC. In fact, the present study shows that higher expression of AR (at mRNA and protein levels) was associated with a better OS (Fig. 1). These findings might seem contradictory to those described previously, but some considerations should be taken into account. Firstly, in the present study, the quantification of the AR at the protein level was performed as a global measure. In other words, no subcellular fractionation protocol was used to quantify the AR at nucleus, where it can be considered activated. As mentioned above, the presence of the AR at the nucleus is associated with a worse prognosis^20^. Thus, without a subcellular fractionation protocol, no considerations about how much AR is activated can be inferred by the global AR expression. In this sense, the AR-Score would better indicate the presence of AR activation.

Secondly, it has been reported that the activation of the AR is associated with a negative feedback for AR expression. The recruitment of lysine specific demethylase 1 (LSD1) and H3K4 demethylation through AR activation suppressed the expression of the AR^39^. Furthermore, other authors have described an increase of the AR expression (at mRNA and protein levels) in HCC cell cultures free of testosterone and a prevention of the AR increase when testosterone is added^40^. These results agree with the negative correlation identified in the present study between the AR-Score-6/7 with the AR expression at both mRNA and protein levels (Fig. 7). Thus, the higher activation of the AR and the resulting upregulation of oncogenic ARGs seem to promote a negative feedback loop to suppress AR expression.

### Limitations

Some limitations should be considered in the present work. On the one hand, ARGs that are included in the analysis to generate the AR-Scores and subsequently to infer the AR activity have previously been validated in prostate cancer, but not in HCC cell lines. In this regard, new studies using Chip-Seq approaches should be performed to identify specific ARGs in the context of HCC. This would allow a more precise quantification of the AR activity in HCC and its relationship with prognosis.

On the other hand, as mentioned above, the RPPA data from the TCGA indicate global protein expression, without quantifying the amount of protein in each subcellular compartment. This point makes it difficult to fit the results of the AR protein expression with those previously reported in literature. Furthermore, this data did not include the phosphorylation state of the AR which seems to play a role in the AR function and subcellular location^41^. However, inferring the AR activity with the AR-Scores seems to be a suitable tool to investigate the role of this receptor in the prognosis of HCC and the results obtained from its use seem to concur with those reported in the literature.

Finally, there is no available information about the androgen blood levels in this cohort of patients. These data would be of great value to help identify the putative origin of the AR activation (i.e. whether it is ligand-dependent or non-ligand dependent).

## Conclusion

HCC patients with high AR expression (at RNA and protein levels) have a longer median overall survival. Nevertheless, AR activity has a different relationship with prognosis. This may be related to the shift in the expression profile of ARGs among histological grades. The AR activity inferred by the relative expression of upregulated to downregulated ARGs in higher histological grades is associated with a worse prognosis. Overall, the results here are supported by the literature and open the door to new research in this field to define the adequacy of the AR as a new druggable target in HCC.

## Supplementary Information

Supplementary Information.
