# Supplementary material for: The androgen receptor expression and its activity have different relationships with prognosis in hepatocellular carcinoma
Source: Sci Rep. 2020 Dec 16;10:22046. doi: 10.1038/s41598-020-79177-2 (PMC7744520; doi:10.1038/s41598-020-79177-2)
Supplement: Supplementary file 1 — Supplementary Information. [file 41598_2020_79177_MOESM1_ESM.docx]

**Title**

The Androgen Receptor Expression and its Activity Have Different Relationships with Prognosis in Hepatocellular Carcinoma.

**Authors**

Acosta-Lopez S^1^, Diaz-Bethencourt D^1^, Concepción-Massip T^2^, Martin-Fernandez de Basoa MC^2^, Plata-Bello A^3^, Gonzalez- Rodriguez A^1^, Perez-Hernandez F^1^, Plata-Bello J^4^.

**Affiliation**

1 Hospital Universitario Nuestra Señora de Candelaria (Liver Unit), S/C de Tenerife, Spain. CP 38010.

2 Hospital Universitario Nuestra Señora de Candelaria (Hormone section of the Biochemical Laboratory), S/C de Tenerife, Spain. CP 38010.

3 Hospital Universitario de Canarias (Department of Urology and Androdology), S/C de Tenerife, Spain. CP 38320.

4 Hospital Universitario de Canarias (Department of Neuroscience), S/C de Tenerife, Spain. CP 38320.

**Running title**

Androgen Receptor and Hepatocellular Carcinoma.

**Corresponding author**

Julio Plata Bello

Address: Hospital Universitario de Canarias (Neuroscience department), Calle Ofra s/n La Cuesta. CP 38320. La Laguna, S/C de Tenerife. Spain. Phone number: +34 922 255 544 / +34 646 625 973. E – mail address: jplata5@hotmail.com

**Keywords**

Hepatocellular carcinoma; Androgen receptor; Androgen pathway.

| **Supplementary table 1. Clinical and molecular features of patients included in the study.** | |
| --- | --- |
|  | **TCGA Patients**  **(n=337)** |
| **Age** | 59.42 (SD=13.0) |
| **Gender (female:male)** | 107:230 |
| **Risk factors (RF)** |  |
| *No history of RF* | 82 (25.8%) |
| *Alcohol* | 107 (33.6%) |
| *HBV infection* | 75 (23.6%) |
| *HCV infection* | 30 (9.4%) |
| *Other* | 15 (4.7%) |
| **Child – Pugh** |  |
| *Grade A* | 201 (90.5%) |
| *Grade B* | 20 (9.0%) |
| *Grade C* | 1 (0.5%) |
| **Specimen collection method** |  |
| *Segmentectomy (single)* | 80 (23.9%) |
| *Segmentectomy (multiple)* | 78 (23.3%) |
| *Lebectomy* | 129 (38.5%) |
| *Extended Lobectomy* | 23 (6.9%) |
| *Other* | 25 (7.5%) |
| **Ishak Score** |  |
| *No fibrosis* | 67 (34.5%%) |
| *Portal fibrosis* | 29 (14.9%) |
| *Fibrous septa* | 25 (12.9%) |
| *Nodular formation and incomplete cirrhosis* | 8 (4.1%) |
| *Established cirrhosis* | 65 (33.5%) |
| **Histologic grade** |  |
| *G1* | 45 (13.4%) |
| *G2* | 162 (48.4%) |
| *G3* | 116 (34.6%) |
| *G4* | 12 (3.6%) |
| **Pathologic stage** |  |
| *Stage I* | 167 (49.6%) |
| *Stage II* | 86 (25.5%) |
| *Stage IIIa-c* | 84 (24.9%) |
| **Vascular invasion** |  |
| *None* | 187 (65.2%) |
| *Micro* | 86 (25.5%) |
| *Macro* | 16 (5.6%) |
| **Residual tumor** |  |
| *R0* | 299 (90.6%) |
| *R1* | 12 (3.6%) |
| *R2* | - |
| *Rx* | 19 (5.8%) |
| **Mutation Count** | 98.66 (SD=109.7) |
| **Fraction Genome Altered** | 0.28 (SD=0.2) |
| **Aneuploidy Score** | 10.61 (SD=8.0) |
| **AR RNA (RPKM)** | 257.95 (SD=381.0) |
| **AR protein (Z)** | -0.01 (SD=0.30) |
| **AR-Score-21 (Hieronimus et al, 2006)** | -0.03 (SD=5.5) |
| **AR-Score-13 (Bolton et al, 2007)** | -0.04 (SD=3.8) |
| SD: Standard Deviation | |

**Supplementary table 2.** Top-10 cancer-related mutated genes in the TCGA whole cohort (363 profiled samples).

|  | ***Number of mutations*** | ***Number of samples with one or more mutations*** | ***Frequency*** |
| --- | --- | --- | --- |
| **TP53** | 113 | 110 | 30.30% |
| **CTNNB1** | 100 | 95 | 26.20% |
| **ALB** | 55 | 47 | 12.90% |
| **PCLO** | 51 | 39 | 10.70% |
| **LRP1B** | 33 | 33 | 9.10% |
| **ARID1A** | 31 | 29 | 8.00% |
| **AXIN1** | 26 | 25 | 6.90% |
| **PRKDC** | 25 | 24 | 6.60% |
| **KMT2D** | 21 | 20 | 5.50% |
| **BAP1** | 22 | 20 | 5.50% |

**Supplementary table 3.** Top-10 cancer-related genes with Copy Number Alteration in the TCGA whole cohort (363 profiled samples).

|  | **Cytoband** | **CNA** | **Number of samples with one or more CNA** | **Frequency** |
| --- | --- | --- | --- | --- |
| **MUC1** | 9p21.3 | 1q22 | AMP | 11.80% |
| **CKS1B** | 9p21.3 | 1q21.3 | AMP | 11.50% |
| **LMNA** | 9p21.3 | 1q22 | AMP | 11.50% |
| **MCL1** | 7p11.2 | 1q21.2 | AMP | 11.50% |
| **RIT1** | 12q14.1 | 1q22 | AMP | 11.50% |
| **ARNT** | 9p21.3 | 1q21.3 | AMP | 11.30% |
| **EXT1** | 4q12 | 8q24.11 | AMP | 11.30% |
| **MYC** | 4q12 | 8q24.21 | AMP | 11.30% |
| **YY1AP1** | 4q12 | 1q22 | AMP | 11.30% |
| **TPM3** | 10q23.31 | 1q21.3 | AMP | 11.00% |

|  | | | |
| --- | --- | --- | --- |
| Supplementary table 4. Comparative analysis of clinical and molecular features between low Androgen Receptor-RNA expression (<=p50) and high Androgen Receptor-RNA expression patients. | | | |
|  | **Low AR**  **(n=169)** | **High AR**  **(n=168)** | **p-value** |
| *Age (years)* | 57.3 (SD=13.3) | 61.6 (SD=12.3) | **.001** |
| *Gender (female:male)* | 64:105 | 43:125 | **.019** |
| *Risk Factors* |  |  | .222 |
| *No risk factors* | 43 (27.4%) | 39 (24.2%) |  |
| *Alcohol consumption* | 57 (36.3%) | 50 (31.1%) |  |
| *Hepatitis B virus* | 35 (22.3%) | 40 (24.8%) |  |
| *Hepatitis C virus* | 15 (9.6%) | 15 (9.3%) |  |
| *NAFLD* | 1 (0.6%) | 8 (5%) |  |
| *Other* | 6 (3.8%) | 9 (5.6%) |  |
| *Child Pugh* |  |  | .62 |
| *A* | 95 (90.5%) | 106 (90.6%) |  |
| *B* | 10 (9.5%) | 10 (8.5%) |  |
| *C* | - | 1 (0.9%) |  |
| *Collection Methods* |  |  | .171 |
| *Single segmentectomy* | 33 (19.6%) | 47 (28.1%) |  |
| *Multiple segmentectomy* | 35 (20.8%) | 43 (25.7%) |  |
| *Lobectomy* | 72 (42.9%) | 57 (34.1%) |  |
| *Lobectomía ampliada* | 13 (7.7%) | 10 (6%) |  |
| *Other* | 15 (8.9%) | 10 (6%) |  |
| *Ishak Score* |  |  | .542 |
| *No fibrosis* | 25 (30.1%) | 42 (37.8%) |  |
| *Portal fibrosis* | 15 (18.1%) | 14 (12.6%) |  |
| *Fibrous septa* | 11 (13.3%) | 14 (12.6%) |  |
| *Nodular formation and incomplete cirrhosis* | 5 (6%) | 3 (2.7%) |  |
| *Established cirrhosis* | 27 (32.5%) | 38 (34.2%) |  |
| *Histologic Grading* |  |  | **.002** |
| *G1* | 14 (8.3%) | 31 (18.7%) |  |
| *G2* | 75 (44.4%) | 87 (52.4%) |  |
| *G3* | 72 (42.6%) | 44 (26.5%) |  |
| *G4* | 8 (4.7%) | 4 (2.4%) |  |
| *Pathologic Stage* |  |  | .335 |
| *Stage I* | 77 (45.6%) | 90 (53.6%) |  |
| *Stage II* | 46 (27.2%) | 40 (23.8%) |  |
| *Stage IIIa-c* | 46 (27.2%) | 38 (22.6%) |  |
| *Vascular invasion* |  |  | **.014** |
| *None* | 84 (59.2%) | 103 (71%) |  |
| *Micro* | 45 (31.7%) | 39 (26.9%) |  |
| *Macro* | 13 (9.2%) | 3 (2.1%) |  |
| *Residual Tumour* |  |  | .423 |
| *R0* | 147 (88.6%) | 152 (92.7%) |  |
| *R1* | 7 (4.2%) | 5 (3%) |  |
| *R2* | - | - |  |
| *Rx* | 12 (7.2%) | 7 (4.3%) |  |
| *Mutation Count* | 87.0 (SD=52.2) | 110.4 (SD=145.7) | .204 |
| *Fraction Genome Altered* | 0.3 (SD=0.2) | 0.25 (SD=0.2) | **.0002** |
| *Aneuploidy Score* | 12.8 (SD=8.8) | 8.3 (SD=6.3) | **.000002** |
| *AR-CVN* |  |  | - |
| *No* | 167 (100%) | 165 (100%) |  |
| *Amplification* | - | - |  |
| *Deep deletion* | - | - |  |
| *AR-Mutation* |  |  | .602 |
| *Putative driver* | - | - |  |
| *Putative passenger* | 2 (1.2%) | 1 (0.6%) |  |
| *No mutation* | 164 (98.8%) | 167 (99.4%) |  |
| *Progression Free Survival* | 17.6 [2.2 – 33.1] | 25.5 [19.3 – 31.8] | .192 |
| *Overall Survival* | 55.4 [26.9 – 83.8] | 80.7 [48.7 – 91.4] | **.045** |
| ^1^ Mann – Whitney U.  ^2^ Fisher exact test.  ^3^ Chi Square.  ^4^ Log Rank test. | | | |

**Supplementary table 5.** GISTIC analysis of the hepatocellular carcinoma top-10 most common focal amplifications and deletions in cancer-related genes for low and high AR - RNA expression groups.

|  | | **AR - RNA expression** | | | |  |  |
| --- | --- | --- | --- | --- | --- | --- | --- |
|  |  | **Low** | | **High** | |  |  |
|  |  | *Count* | *%* | *Count* | *%* | *p-value* | *FDR* |
| MUC1 | No | 147 | 88,0% | 151 | 91,5% | .366 | .527 |
|  | Amplification | 20 | 12,0% | 14 | 8,5% |  |  |
|  | Deep Deletion | 0 | 0,0% | 0 | 0,0% |  |  |
| CKS1B | No | 148 | 88,6% | 151 | 91,5% | .464 | .527 |
|  | Amplification | 19 | 11,4% | 14 | 8,5% |  |  |
|  | Deep Deletion | 0 | 0,0% | 0 | 0,0% |  |  |
| LMNA | No | 147 | 88,0% | 150 | 90,9% | .475 | .527 |
|  | Amplification | 20 | 12,0% | 15 | 9,1% |  |  |
|  | Deep Deletion | 0 | 0,0% | 0 | 0,0% |  |  |
| MCL1 | No | 146 | 87,4% | 151 | 91,5% | .284 | .527 |
|  | Amplification | 21 | 12,6% | 14 | 8,5% |  |  |
|  | Deep Deletion | 0 | 0,0% | 0 | 0,0% |  |  |
| RIT1 | No | 147 | 88,0% | 150 | 90,9% | .475 | .527 |
|  | Amplification | 20 | 12,0% | 15 | 9,1% |  |  |
|  | Deep Deletion | 0 | 0,0% | 0 | 0,0% |  |  |
| ARNT | No | 146 | 87,4% | 151 | 91,5% | .284 | .527 |
|  | Amplification | 21 | 12,6% | 14 | 8,5% |  |  |
|  | Deep Deletion | 0 | 0,0% | 0 | 0,0% |  |  |
| EXT1 | No | 140 | 83,8% | 152 | 92,1% | **.027** | .135 |
|  | Amplification | 27 | 16,2% | 12 | 7,3% |  |  |
|  | Deep Deletion | 0 | 0,0% | 1 | ,6% |  |  |
| MYC | No | 138 | 82,6% | 153 | 92,7% | **.007** | **.070** |
|  | Amplification | 29 | 17,4% | 11 | 6,7% |  |  |
|  | Deep Deletion | 0 | 0,0% | 1 | ,6% |  |  |
| YY1AP1 | No | 148 | 88,6% | 150 | 90,9% | .588 | .588 |
|  | Amplification | 19 | 11,4% | 15 | 9,1% |  |  |
|  | Deep Deletion | 0 | 0,0% | 0 | 0,0% |  |  |
| TPM3 | No | 146 | 87,4% | 152 | 92,1% | .205 | .527 |
|  | Amplification | 21 | 12,6% | 13 | 7,9% |  |  |
|  | Deep Deletion | 0 | 0,0% | 0 | 0,0% |  |  |

**Supplementary table 6.** Mutational signature of the hepatocellular carcinoma top-10 most common cancer-related mutated genes for low and high AR – RNA expression groups.

|  | | **AR – RNA** | | | |  |  |
| --- | --- | --- | --- | --- | --- | --- | --- |
|  |  | **Low** | | **High** | |  |  |
|  |  | *Count* | *%* | *Count* | *%* | *p-value* | *FDR* |
| TP53 | Missense Mutation (putative driver) | 34 | 20,4% | 25 | 15,2% | .187 | .374 |
|  | Truncating Mutation (putative driver) | 21 | 12,6% | 18 | 10,9% |  |  |
|  | Inframe Mutation (putative driver) | 0 | 0,0% | 3 | 1,8% |  |  |
|  | Missense Mutation (putative passenger) | 0 | 0,0% | 0 | 0,0% |  |  |
|  | Truncating Mutation (putative passenger) | 0 | 0,0% | 0 | 0,0% |  |  |
|  | Inframe Mutation (putative passenger) | 0 | 0,0% | 0 | 0,0% |  |  |
|  | No mutation | 112 | 67,1% | 119 | 72,1% |  |  |
| CTNNB1 | Missense Mutation (putative driver) | 30 | 18,0% | 55 | 33,3% | **.003** | **.03** |
|  | Truncating Mutation (putative driver) | 0 | 0,0% | 0 | 0,0% |  |  |
|  | Inframe Mutation (putative driver) | 0 | 0,0% | 1 | ,6% |  |  |
|  | Missense Mutation (putative passenger) | 4 | 2,4% | 0 | 0,0% |  |  |
|  | Truncating Mutation (putative passenger) | 0 | 0,0% | 1 | ,6% |  |  |
|  | Inframe Mutation (putative passenger) | 0 | 0,0% | 0 | 0,0% |  |  |
|  | No mutation | 133 | 79,6% | 108 | 65,5% |  |  |
| ALB | Missense Mutation (putative driver) | 0 | 0,0% | 0 | 0,0% | .855 | .855 |
|  | Truncating Mutation (putative driver) | 0 | 0,0% | 0 | 0,0% |  |  |
|  | Inframe Mutation (putative driver) | 0 | 0,0% | 0 | 0,0% |  |  |
|  | Missense Mutation (putative passenger) | 6 | 3,6% | 6 | 3,6% |  |  |
|  | Truncating Mutation (putative passenger) | 12 | 7,2% | 15 | 9,1% |  |  |
|  | Inframe Mutation (putative passenger) | 1 | ,6% | 2 | 1,2% |  |  |
|  | No mutation | 148 | 88,6% | 142 | 86,1% |  |  |
| PCLO | Missense Mutation (putative driver) | 0 | 0,0% | 0 | 0,0% | .25 | .416 |
|  | Truncating Mutation (putative driver) | 0 | 0,0% | 0 | 0,0% |  |  |
|  | Inframe Mutation (putative driver) | 0 | 0,0% | 0 | 0,0% |  |  |
|  | Missense Mutation (putative passenger) | 15 | 9,0% | 15 | 9,1% |  |  |
|  | Truncating Mutation (putative passenger) | 1 | ,6% | 5 | 3,0% |  |  |
|  | Inframe Mutation (putative passenger) | 0 | 0,0% | 0 | 0,0% |  |  |
|  | No mutation | 151 | 90,4% | 145 | 87,9% |  |  |
| LRP1B | Missense Mutation (putative driver) | 0 | 0,0% | 0 | 0,0% | .073 | .182 |
|  | Truncating Mutation (putative driver) | 0 | 0,0% | 0 | 0,0% |  |  |
|  | Inframe Mutation (putative driver) | 0 | 0,0% | 0 | 0,0% |  |  |
|  | Missense Mutation (putative passenger) | 8 | 4,8% | 17 | 10,3% |  |  |
|  | Truncating Mutation (putative passenger) | 4 | 2,4% | 1 | ,6% |  |  |
|  | Inframe Mutation (putative passenger) | 0 | 0,0% | 0 | 0,0% |  |  |
|  | No mutation | 155 | 92,8% | 147 | 89,1% |  |  |
| ARID1A | Missense Mutation (putative driver) | 0 | 0,0% | 1 | ,6% | .658 | .822 |
|  | Truncating Mutation (putative driver) | 11 | 6,6% | 8 | 4,8% |  |  |
|  | Inframe Mutation (putative driver) | 0 | 0,0% | 0 | 0,0% |  |  |
|  | Missense Mutation (putative passenger) | 4 | 2,4% | 3 | 1,8% |  |  |
|  | Truncating Mutation (putative passenger) | 0 | 0,0% | 0 | 0,0% |  |  |
|  | Inframe Mutation (putative passenger) | 0 | 0,0% | 0 | 0,0% |  |  |
|  | No mutation | 152 | 91,0% | 153 | 92,7% |  |  |
| AXIN1 | Missense Mutation (putative driver) | 0 | 0,0% | 0 | 0,0% | .333 | .475 |
|  | Truncating Mutation (putative driver) | 15 | 9,0% | 8 | 4,8% |  |  |
|  | Inframe Mutation (putative driver) | 0 | 0,0% | 0 | 0,0% |  |  |
|  | Missense Mutation (putative passenger) | 1 | ,6% | 1 | ,6% |  |  |
|  | Truncating Mutation (putative passenger) | 0 | 0,0% | 0 | 0,0% |  |  |
|  | Inframe Mutation (putative passenger) | 0 | 0,0% | 0 | 0,0% |  |  |
|  | No mutation | 151 | 90,4% | 156 | 94,5% |  |  |
| PRKDC | Missense Mutation (putative driver) | 0 | 0,0% | 0 | 0,0% | .052 | .173 |
|  | Truncating Mutation (putative driver) | 0 | 0,0% | 0 | 0,0% |  |  |
|  | Inframe Mutation (putative driver) | 0 | 0,0% | 0 | 0,0% |  |  |
|  | Missense Mutation (putative passenger) | 2 | 1,2% | 11 | 6,7% |  |  |
|  | Truncating Mutation (putative passenger) | 4 | 2,4% | 5 | 3,0% |  |  |
|  | Inframe Mutation (putative passenger) | 1 | ,6% | 0 | 0,0% |  |  |
|  | No mutation | 160 | 95,8% | 149 | 90,3% |  |  |
| KMT2D | Missense Mutation (putative driver) | 0 | 0,0% | 0 | 0,0% | .83 | .855 |
|  | Truncating Mutation (putative driver) | 3 | 1,8% | 3 | 1,8% |  |  |
|  | Inframe Mutation (putative driver) | 0 | 0,0% | 0 | 0,0% |  |  |
|  | Missense Mutation (putative passenger) | 5 | 3,0% | 7 | 4,2% |  |  |
|  | Truncating Mutation (putative passenger) | 0 | 0,0% | 0 | 0,0% |  |  |
|  | Inframe Mutation (putative passenger) | 0 | 0,0% | 0 | 0,0% |  |  |
|  | No mutation | 159 | 95,2% | 155 | 93,9% |  |  |
| BAP1 | Missense Mutation (putative driver) | 0 | 0,0% | 1 | ,6% | **.023** | .115 |
|  | Truncating Mutation (putative driver) | 10 | 6,0% | 1 | ,6% |  |  |
|  | Inframe Mutation (putative driver) | 0 | 0,0% | 0 | 0,0% |  |  |
|  | Missense Mutation (putative passenger) | 3 | 1,8% | 1 | ,6% |  |  |
|  | Truncating Mutation (putative passenger) | 0 | 0,0% | 0 | 0,0% |  |  |
|  | Inframe Mutation (putative passenger) | 0 | 0,0% | 0 | 0,0% |  |  |
|  | No mutation | 154 | 92,2% | 162 | 98,2% |  |  |

| Supplementary table 7. Comparative analysis of clinical and molecular features between low Androgen Receptor-protein (<=p50) and high Androgen Receptor-protein patients. | | | |
| --- | --- | --- | --- |
|  | **Low AR**  **(n=79)** | **High AR**  **(n=78)** | **p-value** |
| *Age (years)* | 58.4 (SD=13.8) | 61.6 (SD=12.3) | .105 |
| *Gender (female:male)* | 28:51 | 28:50 | 1 |
| *Risk Factors* |  |  | .537 |
| *No risk factors* | 29 (38.7%) | 25 (33.8%) |  |
| *Alcohol consumption* | 27 (36%) | 27 (36.5%) |  |
| *Hepatitis B virus* | 6 (8%) | 6 (8.1%) |  |
| *Hepatitis C virus* | 6 (8%) | 12 (16.2%) |  |
| *NAFLD* | 3 (4%) | 3 (4.1%) |  |
| *Other* | 4 (5.3%) | 1 (1.4%) |  |
| *Child Pugh* |  |  | 1 |
| *A* | 31 (91.2%) | 40 (88.9%) |  |
| *B* | 3 (8.8%) | 5 (11.1%) |  |
| *C* | - | - |  |
| *Collection Methods* |  |  | .545 |
| *Single segmentectomy* | 11 (13.9%) | 15 (19.7%) |  |
| *Multiple segmentectomy* | 14 (17.7%) | 19 (25%) |  |
| *Lobectomy* | 36 (45.6%) | 27 (35.5%) |  |
| *Other* | 11 (13.9%) | 8 (10.5%) |  |
| *Ishak Score* |  |  | .327 |
| *No fibrosis* | 18 (52.9%) | 24 (47.1%) |  |
| *Portal fibrosis* | 2 (5.9%) | 11 (21.6%) |  |
| *Fibrous septa* | 3 (8.8%) | 3 (5.9%) |  |
| *Nodular formation and incomplete cirrhosis* | 2 (5.9%) | 1 (2%) |  |
| *Established cirrhosis* | 9 (26.5%) | 12 (23.5%) |  |
| *Histologic Grading* |  |  | .717 |
| *G1* | 12 (15.2%) | 14 (18.2%) |  |
| *G2* | 41 (51.9%) | 37 (48.1%) |  |
| *G3* | 25 (31.6%) | 26 (33.8%) |  |
| *G4* | 1 (1.3%) | - |  |
| *Pathologic Stage* |  |  | .91 |
| *Stage I* | 32 (40.5%) | 31 (39.7%) |  |
| *Stage II* | 19 (24.1%) | 17 (21.8%) |  |
| *Stage IIIa-c* | 28 (35.4%) | 30 (38.5%) |  |
| *Vascular invasion* |  |  | .89 |
| *None* | 41 (66.1%) | 45 (68.2%) |  |
| *Micro* | 18 (29%) | 17 (25.8%) |  |
| *Macro* | 3 (4.8%) | 4 (6.1%) |  |
| *Residual Tumour* |  |  | .533 |
| *R0* | 64 (83.1%) | 66 (89.2%) |  |
| *R1* | 4 (5.2%) | 3 (4.1%) |  |
| *R2* | - | - |  |
| *Rx* | 9 (11.7%) | 5 (6.8%) |  |
| *Mutation Count* | 70.9 (SD=41.5) | 97.9 (SD=105.2) | .143 |
| *Fraction Genome Altered* | 0.3 (SD=0.2) | 0.26 (SD=0.2) | .103 |
| *Aneuploidy Score* | 11.7 (SD=8.1) | 11.2 (SD=9.7) | .298 |
| *AR-CVN* |  |  | - |
| *No* | 77 (100%) | 76 (100%) |  |
| *Amplification* | - | - |  |
| *Deep deletion* | - | - |  |
| *AR-Mutation* |  |  | .219 |
| *Putative driver* | - | - |  |
| *Putative passenger* | 1 (1.3%) | 2 (2.6%) |  |
| *No mutation* | 77 (98.7%) | 74 (97.4%) |  |
| *Progression Free Survival* | 15.6 [7.6 – 23.6] | 18.6 [14.1 – 23.2] | .230 |
| *Overall Survival* | 21.3 [3.8 – 38.9] | 53.3 [40.6 – 66.0] | **.020** |
| ^1^ Mann – Whitney U.  ^2^ Fisher exact test.  ^3^ Chi Square.  ^4^ Log Rank test. | | | |

**Supplementary table 8.** GISTIC analysis of the hepatocellular carcinoma top-10 most common focal amplifications and deletions in cancer-related genes for low and high AR - Protein expression groups.

|  | | **AR - Protein expression** | | | |  |  |
| --- | --- | --- | --- | --- | --- | --- | --- |
|  |  | **Low** | | **High** | |  |  |
|  |  | *Count* | *%* | *Count* | *%* | *p-value* | *FDR* |
| MUC1 | No | 69 | 89,6% | 66 | 86,8% | .625 | .694 |
|  | Amplification | 8 | 10,4% | 10 | 13,2% |  |  |
|  | Deep Deletion | 0 | 0,0% | 0 | 0,0% |  |  |
| CKS1B | No | 70 | 90,9% | 66 | 86,8% | .453 | .694 |
|  | Amplification | 7 | 9,1% | 10 | 13,2% |  |  |
|  | Deep Deletion | 0 | 0,0% | 0 | 0,0% |  |  |
| LMNA | No | 69 | 89,6% | 65 | 85,5% | .473 | .694 |
|  | Amplification | 8 | 10,4% | 11 | 14,5% |  |  |
|  | Deep Deletion | 0 | 0,0% | 0 | 0,0% |  |  |
| MCL1 | No | 69 | 89,6% | 66 | 86,8% | .625 | .694 |
|  | Amplification | 8 | 10,4% | 10 | 13,2% |  |  |
|  | Deep Deletion | 0 | 0,0% | 0 | 0,0% |  |  |
| RIT1 | No | 69 | 89,6% | 65 | 85,5% | .473 | .694 |
|  | Amplification | 8 | 10,4% | 11 | 14,5% |  |  |
|  | Deep Deletion | 0 | 0,0% | 0 | 0,0% |  |  |
| ARNT | No | 68 | 88,3% | 66 | 86,8% | .811 | .811 |
|  | Amplification | 9 | 11,7% | 10 | 13,2% |  |  |
|  | Deep Deletion | 0 | 0,0% | 0 | 0,0% |  |  |
| EXT1 | No | 68 | 88,3% | 67 | 88,2% | .589 | .694 |
|  | Amplification | 8 | 10,4% | 9 | 11,8% |  |  |
|  | Deep Deletion | 1 | 1,3% | 0 | 0,0% |  |  |
| MYC | No | 67 | 87,0% | 66 | 86,8% | .59 | .694 |
|  | Amplification | 9 | 11,7% | 10 | 13,2% |  |  |
|  | Deep Deletion | 1 | 1,3% | 0 | 0,0% |  |  |
| YY1AP1 | No | 70 | 90,9% | 66 | 86,8% | .453 | .694 |
|  | Amplification | 7 | 9,1% | 10 | 13,2% |  |  |
|  | Deep Deletion | 0 | 0,0% | 0 | 0,0% |  |  |
| TPM3 | No | 69 | 89,6% | 66 | 86,8% | .625 | .694 |
|  | Amplification | 8 | 10,4% | 10 | 13,2% |  |  |
|  | Deep Deletion | 0 | 0,0% | 0 | 0,0% |  |  |

**Supplementary table 9.** Mutational signature of the hepatocellular carcinoma top-10 most common cancer-related mutated genes for low and high AR – Protein expression groups.

|  | | **AR – Protein expression** | | | |  |  |
| --- | --- | --- | --- | --- | --- | --- | --- |
|  |  | **Low** | | **High** | |  |  |
|  |  | *Count* | *%* | *Count* | *%* | *p-value* | *FDR* |
| TP53 | Missense Mutation (putative driver) | 12 | 15,6% | 13 | 17,1% | .963 | .963 |
|  | Truncating Mutation (putative driver) | 9 | 11,7% | 7 | 9,2% |  |  |
|  | Inframe Mutation (putative driver) | 1 | 1,3% | 1 | 1,3% |  |  |
|  | Missense Mutation (putative passenger) | 0 | 0,0% | 0 | 0,0% |  |  |
|  | Truncating Mutation (putative passenger) | 0 | 0,0% | 0 | 0,0% |  |  |
|  | Inframe Mutation (putative passenger) | 0 | 0,0% | 0 | 0,0% |  |  |
|  | No mutation | 55 | 71,4% | 55 | 72,4% |  |  |
| CTNNB1 | Missense Mutation (putative driver) | 11 | 14,3% | 21 | 27,6% | .117 | .585 |
|  | Truncating Mutation (putative driver) | 0 | 0,0% | 0 | 0,0% |  |  |
|  | Inframe Mutation (putative driver) | 0 | 0,0% | 1 | 1,3% |  |  |
|  | Missense Mutation (putative passenger) | 1 | 1,3% | 2 | 2,6% |  |  |
|  | Truncating Mutation (putative passenger) | 0 | 0,0% | 0 | 0,0% |  |  |
|  | Inframe Mutation (putative passenger) | 0 | 0,0% | 0 | 0,0% |  |  |
|  | No mutation | 65 | 84,4% | 52 | 68,4% |  |  |
| ALB | Missense Mutation (putative driver) | 0 | 0,0% | 0 | 0,0% | .495 | .756 |
|  | Truncating Mutation (putative driver) | 0 | 0,0% | 0 | 0,0% |  |  |
|  | Inframe Mutation (putative driver) | 0 | 0,0% | 0 | 0,0% |  |  |
|  | Missense Mutation (putative passenger) | 3 | 3,9% | 1 | 1,3% |  |  |
|  | Truncating Mutation (putative passenger) | 4 | 5,2% | 6 | 7,9% |  |  |
|  | Inframe Mutation (putative passenger) | 1 | 1,3% | 0 | 0,0% |  |  |
|  | No mutation | 69 | 89,6% | 69 | 90,8% |  |  |
| PCLO | Missense Mutation (putative driver) | 0 | 0,0% | 0 | 0,0% | .6 | .756 |
|  | Truncating Mutation (putative driver) | 0 | 0,0% | 0 | 0,0% |  |  |
|  | Inframe Mutation (putative driver) | 0 | 0,0% | 0 | 0,0% |  |  |
|  | Missense Mutation (putative passenger) | 6 | 7,8% | 6 | 7,9% |  |  |
|  | Truncating Mutation (putative passenger) | 0 | 0,0% | 1 | 1,3% |  |  |
|  | Inframe Mutation (putative passenger) | 0 | 0,0% | 0 | 0,0% |  |  |
|  | No mutation | 71 | 92,2% | 69 | 90,8% |  |  |
| LRP1B | Missense Mutation (putative driver) | 0 | 0,0% | 0 | 0,0% | .486 | .756 |
|  | Truncating Mutation (putative driver) | 0 | 0,0% | 0 | 0,0% |  |  |
|  | Inframe Mutation (putative driver) | 0 | 0,0% | 0 | 0,0% |  |  |
|  | Missense Mutation (putative passenger) | 5 | 6,5% | 7 | 9,2% |  |  |
|  | Truncating Mutation (putative passenger) | 0 | 0,0% | 1 | 1,3% |  |  |
|  | Inframe Mutation (putative passenger) | 0 | 0,0% | 0 | 0,0% |  |  |
|  | No mutation | 72 | 93,5% | 68 | 89,5% |  |  |
| ARID1A | Missense Mutation (putative driver) | 0 | 0,0% | 0 | 0,0% | .642 | .756 |
|  | Truncating Mutation (putative driver) | 4 | 5,2% | 5 | 6,6% |  |  |
|  | Inframe Mutation (putative driver) | 0 | 0,0% | 0 | 0,0% |  |  |
|  | Missense Mutation (putative passenger) | 2 | 2,6% | 4 | 5,3% |  |  |
|  | Truncating Mutation (putative passenger) | 0 | 0,0% | 0 | 0,0% |  |  |
|  | Inframe Mutation (putative passenger) | 0 | 0,0% | 0 | 0,0% |  |  |
|  | No mutation | 71 | 92,2% | 67 | 88,2% |  |  |
| AXIN1 | Missense Mutation (putative driver) | 0 | 0,0% | 0 | 0,0% | .681 | .756 |
|  | Truncating Mutation (putative driver) | 4 | 5,2% | 2 | 2,6% |  |  |
|  | Inframe Mutation (putative driver) | 0 | 0,0% | 0 | 0,0% |  |  |
|  | Missense Mutation (putative passenger) | 0 | 0,0% | 0 | 0,0% |  |  |
|  | Truncating Mutation (putative passenger) | 0 | 0,0% | 0 | 0,0% |  |  |
|  | Inframe Mutation (putative passenger) | 0 | 0,0% | 0 | 0,0% |  |  |
|  | No mutation | 73 | 94,8% | 74 | 97,4% |  |  |
| PRKDC | Missense Mutation (putative driver) | 0 | 0,0% | 0 | 0,0% | .073 | .585 |
|  | Truncating Mutation (putative driver) | 0 | 0,0% | 0 | 0,0% |  |  |
|  | Inframe Mutation (putative driver) | 0 | 0,0% | 0 | 0,0% |  |  |
|  | Missense Mutation (putative passenger) | 0 | 0,0% | 3 | 3,9% |  |  |
|  | Truncating Mutation (putative passenger) | 0 | 0,0% | 2 | 2,6% |  |  |
|  | Inframe Mutation (putative passenger) | 0 | 0,0% | 0 | 0,0% |  |  |
|  | No mutation | 77 | 100,0% | 71 | 93,4% |  |  |
| KMT2D | Missense Mutation (putative driver) | 0 | 0,0% | 0 | 0,0% | .358 | .756 |
|  | Truncating Mutation (putative driver) | 0 | 0,0% | 2 | 2,6% |  |  |
|  | Inframe Mutation (putative driver) | 0 | 0,0% | 0 | 0,0% |  |  |
|  | Missense Mutation (putative passenger) | 1 | 1,3% | 1 | 1,3% |  |  |
|  | Truncating Mutation (putative passenger) | 0 | 0,0% | 0 | 0,0% |  |  |
|  | Inframe Mutation (putative passenger) | 0 | 0,0% | 0 | 0,0% |  |  |
|  | No mutation | 76 | 98,7% | 73 | 96,1% |  |  |
| BAP1 | Missense Mutation (putative driver) | 0 | 0,0% | 1 | 1,3% | .393 | .756 |
|  | Truncating Mutation (putative driver) | 3 | 3,9% | 3 | 3,9% |  |  |
|  | Inframe Mutation (putative driver) | 0 | 0,0% | 0 | 0,0% |  |  |
|  | Missense Mutation (putative passenger) | 2 | 2,6% | 0 | 0,0% |  |  |
|  | Truncating Mutation (putative passenger) | 0 | 0,0% | 0 | 0,0% |  |  |
|  | Inframe Mutation (putative passenger) | 0 | 0,0% | 0 | 0,0% |  |  |
|  | No mutation | 72 | 93,5% | 72 | 94,7% |  |  |

| Supplementary table 10. Comparative analysis of clinical and molecular features between low AR-Score-21 expression (<=p50) and high AR-Score-21 expression patients. | | | |
| --- | --- | --- | --- |
|  | **Low AR-Score-21**  **(n=169)** | **High AR-Score-21**  **(n=168)** | **p-value** |
| *Age (years)* | 60.7 (SD=12.1) | 58.1 (SD=13.9) | .159 |
| *Gender (female:male)* | 53:116 | 54:114 | .907 |
| *Risk Factors* |  |  | .886 |
| *No risk factors* | 40 (24.8%) | 42 (26.8%) |  |
| *Alcohol consumption* | 58 (36%) | 49 (31.2%) |  |
| *Hepatitis B virus* | 39 (24.2%) | 36 (22.9%) |  |
| *Hepatitis C virus* | 14 (8.7%) | 16 (10.2%) |  |
| *NAFLD* | 4 (2.5%) | 5 (3.2%) |  |
| *Other* | 6 (3.7%) | 9 (5.7%) |  |
| *Child Pugh* |  |  | .127 |
| *A* | 111 (94.1%) | 90 (86.5%) |  |
| *B* | 7 (5.9%) | 13 (12.5%) |  |
| *C* | - | 1 (1%) |  |
| *Collection Methods* |  |  | .698 |
| *Single segmentectomy* | 42 (25.1%) | 38 (22.6%) |  |
| *Multiple segmentectomy* | 39 (23.4%) | 39 (23.2%) |  |
| *Lobectomy* | 65 (38.9%) | 64 (38.1%) |  |
| *Lobectomía ampliada* | 12 (7.2%) | 11 (6.5%) |  |
| *Other* | 9 (5.4%) | 16 (9.5%) |  |
| *Ishak Score* |  |  | .346 |
| *No fibrosis* | 35 (36.5%) | 32 (32.7%) |  |
| *Portal fibrosis* | 15 (15.6%) | 14 (14.3%) |  |
| *Fibrous septa* | 16 (16.7%) | 9 (9.2%) |  |
| *Nodular formation and incomplete cirrhosis* | 3 (3.1%) | 5 (5.1%) |  |
| *Established cirrhosis* | 27 (28.1%) | 38 (34.2%) |  |
| *Histologic Grading* |  |  | .828 |
| *G1* | 20 (12%) | 25 (14.9%) |  |
| *G2* | 80 (47.9%) | 82 (48.8%) |  |
| *G3* | 61 (36.5%) | 55 (32.7%) |  |
| *G4* | 6 (3.6%) | 6 (3.6%) |  |
| *Pathologic Stage* |  |  | .62 |
| *Stage I* | 88 (52.1%) | 79 (47%) |  |
| *Stage II* | 42 (24.9%) | 44 (26.2%) |  |
| *Stage IIIa-c* | 39 (23.1%) | 45 (26.8%) |  |
| *Vascular invasion* |  |  | .579 |
| *None* | 95 (66%) | 92 (64.3%) |  |
| *Micro* | 43 (29.9%) | 41 (28.7%) |  |
| *Macro* | 6 (4.2%) | 10 (7%) |  |
| *Residual Tumour* |  |  | .224 |
| *R0* | 152 (92.1%) | 147 (89.1%) |  |
| *R1* | 7 (4.2%) | 5 (3%) |  |
| *R2* | - | - |  |
| *Rx* | 6 (3.6%) | 13 (7.9%) |  |
| *Mutation Count* | 98.4 (SD=72.5) | 98.9 (SD=137.7) | **.036** |
| *Fraction Genome Altered* | 0.3 (SD=0.2) | 0.26 (SD=0.2) | **.001** |
| *Aneuploidy Score* | 12 (SD=8.1) | 9.2 (SD=7.7) | **.001** |
| *AR-CVN* |  |  | - |
| *No* | 165 (100%) | 167 (100%) |  |
| *Amplification* | - | - |  |
| *Deep deletion* | - | - |  |
| *AR-Mutation* |  |  | .606 |
| *Putative driver* | - | - |  |
| *Putative passenger* | 1 (0.6%) | 2 (1.2%) |  |
| *No mutation* | 166 (99.4%) | 165 (98.8%) |  |
| *Progression Free Survival* | 28.8 [16.3 – 41.2] | 20.9 [13.9 – 27.9] | .239 |
| *Overall Survival* | 70.1 [44.7 – 95.4] | 58.9 [34.5 – 83.2] | .622 |
| ^1^ Mann – Whitney U.  ^2^ Fisher exact test.  ^3^ Chi Square.  ^4^ Log Rank test. | | | |

**Supplementary table 11.** GISTIC analysis of the hepatocellular carcinoma top-10 most common focal amplifications and deletions in cancer-related genes for low and high AR-Score-21 expression groups.

|  | | **AR-Score-21 expression** | | | |  |  |
| --- | --- | --- | --- | --- | --- | --- | --- |
|  |  | **Low** | | **High** | |  |  |
|  |  | *Count* | *%* | *Count* | *%* | *p-value* | *FDR* |
| MUC1 | No | 149 | 90,3% | 149 | 89,2% | .857 | 1 |
|  | Amplification | 16 | 9,7% | 18 | 10,8% |  |  |
|  | Deep Deletion | 0 | 0,0% | 0 | 0,0% |  |  |
| CKS1B | No | 150 | 90,9% | 149 | 89,2% | .714 | 1 |
|  | Amplification | 15 | 9,1% | 18 | 10,8% |  |  |
|  | Deep Deletion | 0 | 0,0% | 0 | 0,0% |  |  |
| LMNA | No | 149 | 90,3% | 148 | 88,6% | .721 | 1 |
|  | Amplification | 16 | 9,7% | 19 | 11,4% |  |  |
|  | Deep Deletion | 0 | 0,0% | 0 | 0,0% |  |  |
| MCL1 | No | 148 | 89,7% | 149 | 89,2% | 1 | 1 |
|  | Amplification | 17 | 10,3% | 18 | 10,8% |  |  |
|  | Deep Deletion | 0 | 0,0% | 0 | 0,0% |  |  |
| RIT1 | No | 149 | 90,3% | 148 | 88,6% | .721 | 1 |
|  | Amplification | 16 | 9,7% | 19 | 11,4% |  |  |
|  | Deep Deletion | 0 | 0,0% | 0 | 0,0% |  |  |
| ARNT | No | 148 | 89,7% | 149 | 89,2% | 1 | 1 |
|  | Amplification | 17 | 10,3% | 18 | 10,8% |  |  |
|  | Deep Deletion | 0 | 0,0% | 0 | 0,0% |  |  |
| EXT1 | No | 140 | 84,8% | 152 | 91,0% | .101 | 1 |
|  | Amplification | 25 | 15,2% | 14 | 8,4% |  |  |
|  | Deep Deletion | 0 | 0,0% | 1 | ,6% |  |  |
| MYC | No | 142 | 86,1% | 149 | 89,2% | .358 | 1 |
|  | Amplification | 23 | 13,9% | 17 | 10,2% |  |  |
|  | Deep Deletion | 0 | 0,0% | 1 | ,6% |  |  |
| YY1AP1 | No | 149 | 90,3% | 149 | 89,2% | .857 | 1 |
|  | Amplification | 16 | 9,7% | 18 | 10,8% |  |  |
|  | Deep Deletion | 0 | 0,0% | 0 | 0,0% |  |  |
| TPM3 | No | 148 | 89,7% | 150 | 89,8% | 1 | 1 |
|  | Amplification | 17 | 10,3% | 17 | 10,2% |  |  |
|  | Deep Deletion | 0 | 0,0% | 0 | 0,0% |  |  |

**Supplementary table 12.** Mutational signature of the hepatocellular carcinoma top-10 most common cancer-related mutated genes for low and high AR-Score-21 expression groups.

|  | | **AR-Score-21 expression** | | | |  |  |
| --- | --- | --- | --- | --- | --- | --- | --- |
|  |  | **Low** | | **High** | |  |  |
|  |  | *Count* | *%* | *Count* | *%* | *p-value* | *FDR* |
| TP53 | Missense Mutation (putative driver) | 27 | 16,4% | 32 | 19,2% | .403 | .673 |
|  | Truncating Mutation (putative driver) | 24 | 14,5% | 15 | 9,0% |  |  |
|  | Inframe Mutation (putative driver) | 1 | ,6% | 2 | 1,2% |  |  |
|  | Missense Mutation (putative passenger) | 0 | 0,0% | 0 | 0,0% |  |  |
|  | Truncating Mutation (putative passenger) | 0 | 0,0% | 0 | 0,0% |  |  |
|  | Inframe Mutation (putative passenger) | 0 | 0,0% | 0 | 0,0% |  |  |
|  | No mutation | 113 | 68,5% | 118 | 70,7% |  |  |
| CTNNB1 | Missense Mutation (putative driver) | 45 | 27,3% | 40 | 24,0% | .48 | .673 |
|  | Truncating Mutation (putative driver) | 0 | 0,0% | 0 | 0,0% |  |  |
|  | Inframe Mutation (putative driver) | 1 | ,6% | 0 | 0,0% |  |  |
|  | Missense Mutation (putative passenger) | 1 | ,6% | 3 | 1,8% |  |  |
|  | Truncating Mutation (putative passenger) | 1 | ,6% | 0 | 0,0% |  |  |
|  | Inframe Mutation (putative passenger) | 0 | 0,0% | 0 | 0,0% |  |  |
|  | No mutation | 117 | 70,9% | 124 | 74,3% |  |  |
| ALB | Missense Mutation (putative driver) | 0 | 0,0% | 0 | 0,0% | .937 | .937 |
|  | Truncating Mutation (putative driver) | 0 | 0,0% | 0 | 0,0% |  |  |
|  | Inframe Mutation (putative driver) | 0 | 0,0% | 0 | 0,0% |  |  |
|  | Missense Mutation (putative passenger) | 6 | 3,6% | 6 | 3,6% |  |  |
|  | Truncating Mutation (putative passenger) | 14 | 8,5% | 13 | 7,8% |  |  |
|  | Inframe Mutation (putative passenger) | 2 | 1,2% | 1 | ,6% |  |  |
|  | No mutation | 143 | 86,7% | 147 | 88,0% |  |  |
| PCLO | Missense Mutation (putative driver) | 0 | 0,0% | 0 | 0,0% | .334 | .673 |
|  | Truncating Mutation (putative driver) | 0 | 0,0% | 0 | 0,0% |  |  |
|  | Inframe Mutation (putative driver) | 0 | 0,0% | 0 | 0,0% |  |  |
|  | Missense Mutation (putative passenger) | 18 | 10,9% | 12 | 7,2% |  |  |
|  | Truncating Mutation (putative passenger) | 4 | 2,4% | 2 | 1,2% |  |  |
|  | Inframe Mutation (putative passenger) | 0 | 0,0% | 0 | 0,0% |  |  |
|  | No mutation | 143 | 86,7% | 153 | 91,6% |  |  |
| LRP1B | Missense Mutation (putative driver) | 0 | 0,0% | 0 | 0,0% | .307 | .673 |
|  | Truncating Mutation (putative driver) | 0 | 0,0% | 0 | 0,0% |  |  |
|  | Inframe Mutation (putative driver) | 0 | 0,0% | 0 | 0,0% |  |  |
|  | Missense Mutation (putative passenger) | 16 | 9,7% | 9 | 5,4% |  |  |
|  | Truncating Mutation (putative passenger) | 2 | 1,2% | 3 | 1,8% |  |  |
|  | Inframe Mutation (putative passenger) | 0 | 0,0% | 0 | 0,0% |  |  |
|  | No mutation | 147 | 89,1% | 155 | 92,8% |  |  |
| ARID1A | Missense Mutation (putative driver) | 0 | 0,0% | 1 | ,6% | .276 | .673 |
|  | Truncating Mutation (putative driver) | 6 | 3,6% | 13 | 7,8% |  |  |
|  | Inframe Mutation (putative driver) | 0 | 0,0% | 0 | 0,0% |  |  |
|  | Missense Mutation (putative passenger) | 3 | 1,8% | 4 | 2,4% |  |  |
|  | Truncating Mutation (putative passenger) | 0 | 0,0% | 0 | 0,0% |  |  |
|  | Inframe Mutation (putative passenger) | 0 | 0,0% | 0 | 0,0% |  |  |
|  | No mutation | 156 | 94,5% | 149 | 89,2% |  |  |
| AXIN1 | Missense Mutation (putative driver) | 0 | 0,0% | 0 | 0,0% | .539 | .673 |
|  | Truncating Mutation (putative driver) | 14 | 8,5% | 9 | 5,4% |  |  |
|  | Inframe Mutation (putative driver) | 0 | 0,0% | 0 | 0,0% |  |  |
|  | Missense Mutation (putative passenger) | 1 | ,6% | 1 | ,6% |  |  |
|  | Truncating Mutation (putative passenger) | 0 | 0,0% | 0 | 0,0% |  |  |
|  | Inframe Mutation (putative passenger) | 0 | 0,0% | 0 | 0,0% |  |  |
|  | No mutation | 150 | 90,9% | 157 | 94,0% |  |  |
| PRKDC | Missense Mutation (putative driver) | 0 | 0,0% | 0 | 0,0% | .439 | .673 |
|  | Truncating Mutation (putative driver) | 0 | 0,0% | 0 | 0,0% |  |  |
|  | Inframe Mutation (putative driver) | 0 | 0,0% | 0 | 0,0% |  |  |
|  | Missense Mutation (putative passenger) | 5 | 3,0% | 8 | 4,8% |  |  |
|  | Truncating Mutation (putative passenger) | 3 | 1,8% | 6 | 3,6% |  |  |
|  | Inframe Mutation (putative passenger) | 1 | ,6% | 0 | 0,0% |  |  |
|  | No mutation | 156 | 94,5% | 153 | 91,6% |  |  |
| KMT2D | Missense Mutation (putative driver) | 0 | 0,0% | 0 | 0,0% | .137 | .673 |
|  | Truncating Mutation (putative driver) | 4 | 2,4% | 2 | 1,2% |  |  |
|  | Inframe Mutation (putative driver) | 0 | 0,0% | 0 | 0,0% |  |  |
|  | Missense Mutation (putative passenger) | 9 | 5,5% | 3 | 1,8% |  |  |
|  | Truncating Mutation (putative passenger) | 0 | 0,0% | 0 | 0,0% |  |  |
|  | Inframe Mutation (putative passenger) | 0 | 0,0% | 0 | 0,0% |  |  |
|  | No mutation | 152 | 92,1% | 162 | 97,0% |  |  |
| BAP1 | Missense Mutation (putative driver) | 1 | ,6% | 0 | 0,0% | .779 | .865 |
|  | Truncating Mutation (putative driver) | 5 | 3,0% | 6 | 3,6% |  |  |
|  | Inframe Mutation (putative driver) | 0 | 0,0% | 0 | 0,0% |  |  |
|  | Missense Mutation (putative passenger) | 2 | 1,2% | 2 | 1,2% |  |  |
|  | Truncating Mutation (putative passenger) | 0 | 0,0% | 0 | 0,0% |  |  |
|  | Inframe Mutation (putative passenger) | 0 | 0,0% | 0 | 0,0% |  |  |
|  | No mutation | 157 | 95,2% | 159 | 95,2% |  |  |

| Supplementary table 13. Comparative analysis of clinical and molecular features between low AR-Score-13 expression (<=p50) and high AR-Score-13 expression patients. | | | |
| --- | --- | --- | --- |
|  | **Low AR-Score-13**  **(n=169)** | **High AR-Score-13**  **(n=168)** | **p-value** |
| *Age (years)* | 60 (SD=12.1) | 59 (SD=13.8) | .994 |
| *Gender (female:male)* | 51:118 | 56:112 | .56 |
| *Risk Factors* |  |  | .202 |
| *No risk factors* | 36 (22.5%) | 46 (29.1%) |  |
| *Alcohol consumption* | 64 (40%) | 43 (27.2%) |  |
| *Hepatitis B virus* | 38 (23.8%) | 37 (23.4%) |  |
| *Hepatitis C virus* | 12 (7.5%) | 18 (11.4%) |  |
| *NAFLD* | 4 (2.5%) | 5 (3.2%) |  |
| *Other* | 6 (3.8%) | 9 (5.7%) |  |
| *Child Pugh* |  |  | .518 |
| *A* | 105 (91.3%) | 96 (89.7%) |  |
| *B* | 9 (7.8%) | 11 (10.3%) |  |
| *C* | 1 (0.9%) | - |  |
| *Collection Methods* |  |  | **.025** |
| *Single segmentectomy* | 47 (28%) | 33 (19.8%) |  |
| *Multiple segmentectomy* | 39 (23.2%) | 39 (23.4%) |  |
| *Lobectomy* | 68 (40.5%) | 61 (36.5%) |  |
| *Lobectomía ampliada* | 7 (4.2%) | 16 (9.6%) |  |
| *Other* | 7 (4.2%) | 18 (10.8%) |  |
| *Ishak Score* |  |  | .522 |
| *No fibrosis* | 30 (32.3%) | 37 (36.6%) |  |
| *Portal fibrosis* | 16 (17.2%) | 13 (12.9%) |  |
| *Fibrous septa* | 14 (15.1%) | 11 (10.9%) |  |
| *Nodular formation and incomplete cirrhosis* | 2 (2.2%) | 6 (5.9%) |  |
| *Established cirrhosis* | 31 (33.3%) | 34 (33.7%) |  |
| *Histologic Grading* |  |  | **.05** |
| *G1* | 15 (8.9%) | 30 (18%) |  |
| *G2* | 80 (47.6%) | 82 (49.1%) |  |
| *G3* | 67 (39.9%) | 49 (29.3%) |  |
| *G4* | 6 (3.6%) | 6 (3.6%) |  |
| *Pathologic Stage* |  |  | .582 |
| *Stage I* | 86 (50.9%) | 82 (48.2%) |  |
| *Stage II* | 39 (23.1%) | 47 (28%) |  |
| *Stage IIIa-c* | 44 (26%) | 40 (23.8%) |  |
| *Vascular invasion* |  |  | .426 |
| *None* | 97 (68.3%) | 90 (62.1%) |  |
| *Micro* | 39 (27.5%) | 45 (31%) |  |
| *Macro* | 6 (4.2%) | 10 (6.9%) |  |
| *Residual Tumour* |  |  | .116 |
| *R0* | 155 (93.9%) | 144 (87.3%) |  |
| *R1* | 4 (2.4%) | 8 (4.8%) |  |
| *R2* | - | - |  |
| *Rx* | 6 (3.6%) | 13 (7.9%) |  |
| *Mutation Count* | 101 (SD=80.6) | 96.3 (SD=132.9) | **.027** |
| *Fraction Genome Altered* | 0.30 (SD=0.2) | 0.27 (SD=0.2) | **.04** |
| *Aneuploidy Score* | 12 (SD=8.5) | 9.2 (SD=7.1) | **.002** |
| *AR-CVN* |  |  | - |
| *No* | 167 (100%) | 165 (100%) |  |
| *Amplification* | - | - |  |
| *Deep deletion* | - | - |  |
| *AR-Mutation* |  |  | .22 |
| *Putative driver* | - | - |  |
| *Putative passenger* | 3 (1.8%) | - |  |
| *No mutation* | 164 (98.2%) | 167 (100%) |  |
| *AR-Score-21* | -1.6 (SD=5.1) | 1.65 (SD=5.3) | **.0000001** |
| *AR-Score-6* | 0.2 (SD=0.2) | 0.2 (SD=0.3) | .974 |
| *AR-Score-7* | 3.08 (SD=3.0) | 5.1 (SD=7.8) | .861 |
| *Progression Free Survival* | 25.5 [15.9 – 35.1] | 21.0 [9.3 – 32.7] | .493 |
| *Overall Survival* | 80.7 [53.8 – 107.7] | 60.9 [40.8 – 80.9] | .504 |
| ^1^ Mann – Whitney U.  ^2^ Fisher exact test.  ^3^ Chi Square.  ^4^ Log Rank test. | | | |

**Supplementary table 14.** GISTIC analysis of the hepatocellular carcinoma top-10 most common focal amplifications and deletions in cancer-related genes for low and high AR-Score-13 expression groups.

|  | | **AR-Score-13 expression** | | | |  |  |
| --- | --- | --- | --- | --- | --- | --- | --- |
|  |  | **Low** | | **High** | |  |  |
|  |  | *Count* | *%* | *Count* | *%* | *p-value* | *FDR* |
| MUC1 | No | 156 | 93,4% | 142 | 86,1% | **.03** | .037 |
|  | Amplification | 11 | 6,6% | 23 | 13,9% |  |  |
|  | Deep Deletion | 0 | 0,0% | 0 | 0,0% |  |  |
| CKS1B | No | 158 | 94,6% | 141 | 85,5% | **.006** | .022 |
|  | Amplification | 9 | 5,4% | 24 | 14,5% |  |  |
|  | Deep Deletion | 0 | 0,0% | 0 | 0,0% |  |  |
| LMNA | No | 157 | 94,0% | 140 | 84,8% | **.007** | .022 |
|  | Amplification | 10 | 6,0% | 25 | 15,2% |  |  |
|  | Deep Deletion | 0 | 0,0% | 0 | 0,0% |  |  |
| MCL1 | No | 156 | 93,4% | 141 | 85,5% | **.02** | .028 |
|  | Amplification | 11 | 6,6% | 24 | 14,5% |  |  |
|  | Deep Deletion | 0 | 0,0% | 0 | 0,0% |  |  |
| RIT1 | No | 156 | 93,4% | 141 | 85,5% | **.02** | .028 |
|  | Amplification | 11 | 6,6% | 24 | 14,5% |  |  |
|  | Deep Deletion | 0 | 0,0% | 0 | 0,0% |  |  |
| ARNT | No | 157 | 94,0% | 140 | 84,8% | **.007** | .022 |
|  | Amplification | 10 | 6,0% | 25 | 15,2% |  |  |
|  | Deep Deletion | 0 | 0,0% | 0 | 0,0% |  |  |
| EXT1 | No | 143 | 85,6% | 149 | 90,3% | .203 | .225 |
|  | Amplification | 24 | 14,4% | 15 | 9,1% |  |  |
|  | Deep Deletion | 0 | 0,0% | 1 | ,6% |  |  |
| MYC | No | 145 | 86,8% | 146 | 88,5% | .499 | .499 |
|  | Amplification | 22 | 13,2% | 18 | 10,9% |  |  |
|  | Deep Deletion | 0 | 0,0% | 1 | ,6% |  |  |
| YY1AP1 | No | 157 | 94,0% | 141 | 85,5% | **.011** | .022 |
|  | Amplification | 10 | 6,0% | 24 | 14,5% |  |  |
|  | Deep Deletion | 0 | 0,0% | 0 | 0,0% |  |  |
| TPM3 | No | 157 | 94,0% | 141 | 85,5% | **.011** | .022 |
|  | Amplification | 10 | 6,0% | 24 | 14,5% |  |  |
|  | Deep Deletion | 0 | 0,0% | 0 | 0,0% |  |  |

**Supplementary table 15.** Mutational signature of the hepatocellular carcinoma top-10 most common cancer-related mutated genes for low and high AR-Score-13 expression groups.

|  | | **AR-Score-13 expression** | | | |  |  |
| --- | --- | --- | --- | --- | --- | --- | --- |
|  |  | **Low** | | **High** | |  |  |
|  |  | *Count* | *%* | *Count* | *%* | *p-value* | *FDR* |
| TP53 | Missense Mutation (putative driver) | 31 | 18,6% | 28 | 17,0% | .863 | .863 |
|  | Truncating Mutation (putative driver) | 21 | 12,6% | 18 | 10,9% |  |  |
|  | Inframe Mutation (putative driver) | 1 | ,6% | 2 | 1,2% |  |  |
|  | Missense Mutation (putative passenger) | 0 | 0,0% | 0 | 0,0% |  |  |
|  | Truncating Mutation (putative passenger) | 0 | 0,0% | 0 | 0,0% |  |  |
|  | Inframe Mutation (putative passenger) | 0 | 0,0% | 0 | 0,0% |  |  |
|  | No mutation | 114 | 68,3% | 117 | 70,9% |  |  |
| CTNNB1 | Missense Mutation (putative driver) | 48 | 28,7% | 37 | 22,4% | .314 | .731 |
|  | Truncating Mutation (putative driver) | 0 | 0,0% | 0 | 0,0% |  |  |
|  | Inframe Mutation (putative driver) | 1 | ,6% | 0 | 0,0% |  |  |
|  | Missense Mutation (putative passenger) | 1 | ,6% | 3 | 1,8% |  |  |
|  | Truncating Mutation (putative passenger) | 1 | ,6% | 0 | 0,0% |  |  |
|  | Inframe Mutation (putative passenger) | 0 | 0,0% | 0 | 0,0% |  |  |
|  | No mutation | 116 | 69,5% | 125 | 75,8% |  |  |
| ALB | Missense Mutation (putative driver) | 0 | 0,0% | 0 | 0,0% | .332 | .731 |
|  | Truncating Mutation (putative driver) | 0 | 0,0% | 0 | 0,0% |  |  |
|  | Inframe Mutation (putative driver) | 0 | 0,0% | 0 | 0,0% |  |  |
|  | Missense Mutation (putative passenger) | 7 | 4,2% | 5 | 3,0% |  |  |
|  | Truncating Mutation (putative passenger) | 13 | 7,8% | 14 | 8,5% |  |  |
|  | Inframe Mutation (putative passenger) | 0 | 0,0% | 3 | 1,8% |  |  |
|  | No mutation | 147 | 88,0% | 143 | 86,7% |  |  |
| PCLO | Missense Mutation (putative driver) | 0 | 0,0% | 0 | 0,0% | .537 | .767 |
|  | Truncating Mutation (putative driver) | 0 | 0,0% | 0 | 0,0% |  |  |
|  | Inframe Mutation (putative driver) | 0 | 0,0% | 0 | 0,0% |  |  |
|  | Missense Mutation (putative passenger) | 18 | 10,8% | 12 | 7,3% |  |  |
|  | Truncating Mutation (putative passenger) | 3 | 1,8% | 3 | 1,8% |  |  |
|  | Inframe Mutation (putative passenger) | 0 | 0,0% | 0 | 0,0% |  |  |
|  | No mutation | 146 | 87,4% | 150 | 90,9% |  |  |
| LRP1B | Missense Mutation (putative driver) | 0 | 0,0% | 0 | 0,0% | .398 | .731 |
|  | Truncating Mutation (putative driver) | 0 | 0,0% | 0 | 0,0% |  |  |
|  | Inframe Mutation (putative driver) | 0 | 0,0% | 0 | 0,0% |  |  |
|  | Missense Mutation (putative passenger) | 13 | 7,8% | 12 | 7,3% |  |  |
|  | Truncating Mutation (putative passenger) | 4 | 2,4% | 1 | ,6% |  |  |
|  | Inframe Mutation (putative passenger) | 0 | 0,0% | 0 | 0,0% |  |  |
|  | No mutation | 150 | 89,8% | 152 | 92,1% |  |  |
| ARID1A | Missense Mutation (putative driver) | 0 | 0,0% | 1 | ,6% | .75 | .863 |
|  | Truncating Mutation (putative driver) | 9 | 5,4% | 10 | 6,1% |  |  |
|  | Inframe Mutation (putative driver) | 0 | 0,0% | 0 | 0,0% |  |  |
|  | Missense Mutation (putative passenger) | 4 | 2,4% | 3 | 1,8% |  |  |
|  | Truncating Mutation (putative passenger) | 0 | 0,0% | 0 | 0,0% |  |  |
|  | Inframe Mutation (putative passenger) | 0 | 0,0% | 0 | 0,0% |  |  |
|  | No mutation | 154 | 92,2% | 151 | 91,5% |  |  |
| AXIN1 | Missense Mutation (putative driver) | 0 | 0,0% | 0 | 0,0% | .16 | .731 |
|  | Truncating Mutation (putative driver) | 16 | 9,6% | 7 | 4,2% |  |  |
|  | Inframe Mutation (putative driver) | 0 | 0,0% | 0 | 0,0% |  |  |
|  | Missense Mutation (putative passenger) | 1 | ,6% | 1 | ,6% |  |  |
|  | Truncating Mutation (putative passenger) | 0 | 0,0% | 0 | 0,0% |  |  |
|  | Inframe Mutation (putative passenger) | 0 | 0,0% | 0 | 0,0% |  |  |
|  | No mutation | 150 | 89,8% | 157 | 95,2% |  |  |
| PRKDC | Missense Mutation (putative driver) | 0 | 0,0% | 0 | 0,0% | .439 | .731 |
|  | Truncating Mutation (putative driver) | 0 | 0,0% | 0 | 0,0% |  |  |
|  | Inframe Mutation (putative driver) | 0 | 0,0% | 0 | 0,0% |  |  |
|  | Missense Mutation (putative passenger) | 8 | 4,8% | 5 | 3,0% |  |  |
|  | Truncating Mutation (putative passenger) | 3 | 1,8% | 6 | 3,6% |  |  |
|  | Inframe Mutation (putative passenger) | 0 | 0,0% | 1 | ,6% |  |  |
|  | No mutation | 156 | 93,4% | 153 | 92,7% |  |  |
| KMT2D | Missense Mutation (putative driver) | 0 | 0,0% | 0 | 0,0% | .219 | .731 |
|  | Truncating Mutation (putative driver) | 3 | 1,8% | 3 | 1,8% |  |  |
|  | Inframe Mutation (putative driver) | 0 | 0,0% | 0 | 0,0% |  |  |
|  | Missense Mutation (putative passenger) | 9 | 5,4% | 3 | 1,8% |  |  |
|  | Truncating Mutation (putative passenger) | 0 | 0,0% | 0 | 0,0% |  |  |
|  | Inframe Mutation (putative passenger) | 0 | 0,0% | 0 | 0,0% |  |  |
|  | No mutation | 155 | 92,8% | 159 | 96,4% |  |  |
| BAP1 | Missense Mutation (putative driver) | 1 | ,6% | 0 | 0,0% | .779 | .863 |
|  | Truncating Mutation (putative driver) | 5 | 3,0% | 6 | 3,6% |  |  |
|  | Inframe Mutation (putative driver) | 0 | 0,0% | 0 | 0,0% |  |  |
|  | Missense Mutation (putative passenger) | 2 | 1,2% | 2 | 1,2% |  |  |
|  | Truncating Mutation (putative passenger) | 0 | 0,0% | 0 | 0,0% |  |  |
|  | Inframe Mutation (putative passenger) | 0 | 0,0% | 0 | 0,0% |  |  |
|  | No mutation | 159 | 95,2% | 157 | 95,2% |  |  |

| Supplementary table 16. Comparative analysis of clinical and molecular features between low AR-Score-6 expression (<=p50) and high AR-Score-6 expression patients. | | | |
| --- | --- | --- | --- |
|  | **Low AR-Score-6**  **(n=168)** | **High AR-Score-6**  **(n=169)** | **p-value** |
| *Age (years)* | 61.1 (SD=12) | 57.8 (SD=13.7) | **.033** |
| *Gender (female:male)* | 48:120 | 59:110 | .242 |
| *Risk Factors* |  |  | .215 |
| *No risk factors* | 43 (26.7%) | 39 (24.8%) |  |
| *Alcohol consumption* | 51 (31.7%) | 56 (35.7%) |  |
| *Hepatitis B virus* | 36 (22.4%) | 39 (24.8%) |  |
| *Hepatitis C virus* | 17 (10.6%) | 13 (8.3%) |  |
| *NAFLD* | 8 (5%) | 1 (0.6%) |  |
| *Other* | 6 (3.7%) | 9 (5.7%) |  |
| *Child Pugh* |  |  | .155 |
| *A* | 110 (93.2%) | 91 (87.5%) |  |
| *B* | 7 (5.9%) | 13 (12.5%) |  |
| *C* | 1 (0.8%) | - |  |
| *Collection Methods* |  |  | **.012** |
| *Single segmentectomy* | 45 (27.1%) | 35 (20.7%) |  |
| *Multiple segmentectomy* | 49 (29.5%) | 29 (17.2%) |  |
| *Lobectomy* | 54 (32.5%) | 75 (44.4%) |  |
| *Lobectomía ampliada* | 9 (5.4%) | 14 (8.3%) |  |
| *Other* | 9 (5.4%) | 16 (9.5%) |  |
| *Ishak Score* |  |  | .386 |
| *No fibrosis* | 40 (35.7%) | 27 (32.9%) |  |
| *Portal fibrosis* | 12 (10.7%) | 17 (20.7%) |  |
| *Fibrous septa* | 14 (12.5%) | 11 (13.4%) |  |
| *Nodular formation and incomplete cirrhosis* | 5 (4.5%) | 3 (3.7%) |  |
| *Established cirrhosis* | 41 (36.6%) | 24 (29.3%) |  |
| *Histologic Grading* |  |  | **.003** |
| *G1* | 29 (17.4%) | 16 (9.5%) |  |
| *G2* | 90 (53.9%) | 72 (42.9%) |  |
| *G3* | 44 (26.3%) | 72 (42.9%) |  |
| *G4* | 4 (2.4%) | 8 (4.8%) |  |
| *Pathologic Stage* |  |  | **.021** |
| *Stage I* | 96 (57.1%) | 71 (42%) |  |
| *Stage II* | 37 (22%) | 49 (29%) |  |
| *Stage IIIa-c* | 35 (20.8%) | 49 (29%) |  |
| *Vascular invasion* |  |  | .211 |
| *None* | 100 (68.5%) | 87 (61.7%) |  |
| *Micro* | 41 (28.1%) | 43 (30.5%) |  |
| *Macro* | 5 (3.4%) | 11 (7.8%) |  |
| *Residual Tumour* |  |  | .641 |
| *R0* | 152 (92.1%) | 147 (89.1%) |  |
| *R1* | 5 (3%) | 7 (4.2%) |  |
| *R2* | - | - |  |
| *Rx* | 8 (4.8%) | 11 (6.7%) |  |
| *Mutation Count* | 103.6 (SD=136.4) | 93.7 (SD=74.5) | .882 |
| *Fraction Genome Altered* | 0.25 (SD=0.2) | 0.3 (SD=0.2) | **.0002** |
| *Aneuploidy Score* | 9.1 (SD=7.7) | 12.1 (SD=7.9) | **.00004** |
| *AR-CVN* |  |  | - |
| *No* | 165 (100%) | 167 (100%) |  |
| *Amplification* | - | - |  |
| *Deep deletion* | - | - |  |
| *AR-Mutation* |  |  | .221 |
| *Putative driver* | - | - |  |
| *Putative passenger* | 2 (1.2%) | 1 (0.6%) |  |
| *No mutation* | 164 (98.8%) | 167 (99.4%) |  |
| *Progression Free Survival* | 32.5 [14.8 – 50.3] | 16.5 [10.1 – 22.9] | **.005** |
| *Overall Survival* | 83.2 [43.2 – 123.3] | 58.9 [42.7 – 75.1] | .153 |
| ^1^ Mann – Whitney U.  ^2^ Fisher exact test.  ^3^ Chi Square.  ^4^ Log Rank test. | | | |

**Supplementary table 17.** GISTIC analysis of the hepatocellular carcinoma top-10 most common focal amplifications and deletions in cancer-related genes for low and high AR-Score-6 expression groups.

|  | | **AR-Score-6 expression** | | | |  |  |
| --- | --- | --- | --- | --- | --- | --- | --- |
|  |  | **Low** | | **High** | |  |  |
|  |  | *Count* | *%* | *Count* | *%* | *p-value* | *FDR* |
| MUC1 | No | 149 | 90,3% | 149 | 89,2% | .857 | 1 |
|  | Amplification | 16 | 9,7% | 18 | 10,8% |  |  |
|  | Deep Deletion | 0 | 0,0% | 0 | 0,0% |  |  |
| CKS1B | No | 149 | 90,3% | 150 | 89,8% | 1 | 1 |
|  | Amplification | 16 | 9,7% | 17 | 10,2% |  |  |
|  | Deep Deletion | 0 | 0,0% | 0 | 0,0% |  |  |
| LMNA | No | 148 | 89,7% | 149 | 89,2% | 1 | 1 |
|  | Amplification | 17 | 10,3% | 18 | 10,8% |  |  |
|  | Deep Deletion | 0 | 0,0% | 0 | 0,0% |  |  |
| MCL1 | No | 147 | 89,1% | 150 | 89,8% | .86 | 1 |
|  | Amplification | 18 | 10,9% | 17 | 10,2% |  |  |
|  | Deep Deletion | 0 | 0,0% | 0 | 0,0% |  |  |
| RIT1 | No | 148 | 89,7% | 149 | 89,2% | 1 | 1 |
|  | Amplification | 17 | 10,3% | 18 | 10,8% |  |  |
|  | Deep Deletion | 0 | 0,0% | 0 | 0,0% |  |  |
| ARNT | No | 148 | 89,7% | 149 | 89,2% | 1 | 1 |
|  | Amplification | 17 | 10,3% | 18 | 10,8% |  |  |
|  | Deep Deletion | 0 | 0,0% | 0 | 0,0% |  |  |
| EXT1 | No | 148 | 89,7% | 144 | 86,2% | .317 | 1 |
|  | Amplification | 16 | 9,7% | 23 | 13,8% |  |  |
|  | Deep Deletion | 1 | ,6% | 0 | 0,0% |  |  |
| MYC | No | 149 | 90,3% | 142 | 85,0% | .161 | 1 |
|  | Amplification | 15 | 9,1% | 25 | 15,0% |  |  |
|  | Deep Deletion | 1 | ,6% | 0 | 0,0% |  |  |
| YY1AP1 | No | 148 | 89,7% | 150 | 89,8% | 1 | 1 |
|  | Amplification | 17 | 10,3% | 17 | 10,2% |  |  |
|  | Deep Deletion | 0 | 0,0% | 0 | 0,0% |  |  |
| TPM3 | No | 149 | 90,3% | 149 | 89,2% | .857 | 1 |
|  | Amplification | 16 | 9,7% | 18 | 10,8% |  |  |
|  | Deep Deletion | 0 | 0,0% | 0 | 0,0% |  |  |

**Supplementary table 18.** Mutational signature of the hepatocellular carcinoma top-10 most common cancer-related mutated genes for low and high AR-Score-6 expression groups.

|  | | **AR-Score-6 expression** | | | |  |  |
| --- | --- | --- | --- | --- | --- | --- | --- |
|  |  | **Low** | | **High** | |  |  |
|  |  | *Count* | *%* | *Count* | *%* | *p-value* | *FDR* |
| TP53 | Missense Mutation (putative driver) | 22 | 13,3% | 37 | 22,2% | **.063** | .506 |
|  | Truncating Mutation (putative driver) | 16 | 9,7% | 23 | 13,8% |  |  |
|  | Inframe Mutation (putative driver) | 1 | ,6% | 2 | 1,2% |  |  |
|  | Missense Mutation (putative passenger) | 0 | 0,0% | 0 | 0,0% |  |  |
|  | Truncating Mutation (putative passenger) | 0 | 0,0% | 0 | 0,0% |  |  |
|  | Inframe Mutation (putative passenger) | 0 | 0,0% | 0 | 0,0% |  |  |
|  | No mutation | 126 | 76,4% | 105 | 62,9% |  |  |
| CTNNB1 | Missense Mutation (putative driver) | 40 | 24,2% | 45 | 26,9% | **.48** | .685 |
|  | Truncating Mutation (putative driver) | 0 | 0,0% | 0 | 0,0% |  |  |
|  | Inframe Mutation (putative driver) | 0 | 0,0% | 1 | ,6% |  |  |
|  | Missense Mutation (putative passenger) | 1 | ,6% | 3 | 1,8% |  |  |
|  | Truncating Mutation (putative passenger) | 0 | 0,0% | 1 | ,6% |  |  |
|  | Inframe Mutation (putative passenger) | 0 | 0,0% | 0 | 0,0% |  |  |
|  | No mutation | 124 | 75,2% | 117 | 70,1% |  |  |
| ALB | Missense Mutation (putative driver) | 0 | 0,0% | 0 | 0,0% | **.315** | .585 |
|  | Truncating Mutation (putative driver) | 0 | 0,0% | 0 | 0,0% |  |  |
|  | Inframe Mutation (putative driver) | 0 | 0,0% | 0 | 0,0% |  |  |
|  | Missense Mutation (putative passenger) | 6 | 3,6% | 6 | 3,6% |  |  |
|  | Truncating Mutation (putative passenger) | 15 | 9,1% | 12 | 7,2% |  |  |
|  | Inframe Mutation (putative passenger) | 3 | 1,8% | 0 | 0,0% |  |  |
|  | No mutation | 141 | 85,5% | 149 | 89,2% |  |  |
| PCLO | Missense Mutation (putative driver) | 0 | 0,0% | 0 | 0,0% | .702 | .752 |
|  | Truncating Mutation (putative driver) | 0 | 0,0% | 0 | 0,0% |  |  |
|  | Inframe Mutation (putative driver) | 0 | 0,0% | 0 | 0,0% |  |  |
|  | Missense Mutation (putative passenger) | 15 | 9,1% | 15 | 9,0% |  |  |
|  | Truncating Mutation (putative passenger) | 4 | 2,4% | 2 | 1,2% |  |  |
|  | Inframe Mutation (putative passenger) | 0 | 0,0% | 0 | 0,0% |  |  |
|  | No mutation | 146 | 88,5% | 150 | 89,8% |  |  |
| LRP1B | Missense Mutation (putative driver) | 0 | 0,0% | 0 | 0,0% | .333 | .585 |
|  | Truncating Mutation (putative driver) | 0 | 0,0% | 0 | 0,0% |  |  |
|  | Inframe Mutation (putative driver) | 0 | 0,0% | 0 | 0,0% |  |  |
|  | Missense Mutation (putative passenger) | 11 | 6,7% | 14 | 8,4% |  |  |
|  | Truncating Mutation (putative passenger) | 1 | ,6% | 4 | 2,4% |  |  |
|  | Inframe Mutation (putative passenger) | 0 | 0,0% | 0 | 0,0% |  |  |
|  | No mutation | 153 | 92,7% | 149 | 89,2% |  |  |
| ARID1A | Missense Mutation (putative driver) | 1 | ,6% | 0 | 0,0% | .658 | .752 |
|  | Truncating Mutation (putative driver) | 8 | 4,8% | 11 | 6,6% |  |  |
|  | Inframe Mutation (putative driver) | 0 | 0,0% | 0 | 0,0% |  |  |
|  | Missense Mutation (putative passenger) | 3 | 1,8% | 4 | 2,4% |  |  |
|  | Truncating Mutation (putative passenger) | 0 | 0,0% | 0 | 0,0% |  |  |
|  | Inframe Mutation (putative passenger) | 0 | 0,0% | 0 | 0,0% |  |  |
|  | No mutation | 153 | 92,7% | 152 | 91,0% |  |  |
| AXIN1 | Missense Mutation (putative driver) | 0 | 0,0% | 0 | 0,0% | .126 | .506 |
|  | Truncating Mutation (putative driver) | 8 | 4,8% | 15 | 9,0% |  |  |
|  | Inframe Mutation (putative driver) | 0 | 0,0% | 0 | 0,0% |  |  |
|  | Missense Mutation (putative passenger) | 2 | 1,2% | 0 | 0,0% |  |  |
|  | Truncating Mutation (putative passenger) | 0 | 0,0% | 0 | 0,0% |  |  |
|  | Inframe Mutation (putative passenger) | 0 | 0,0% | 0 | 0,0% |  |  |
|  | No mutation | 155 | 93,9% | 152 | 91,0% |  |  |
| PRKDC | Missense Mutation (putative driver) | 0 | 0,0% | 0 | 0,0% | .752 | .752 |
|  | Truncating Mutation (putative driver) | 0 | 0,0% | 0 | 0,0% |  |  |
|  | Inframe Mutation (putative driver) | 0 | 0,0% | 0 | 0,0% |  |  |
|  | Missense Mutation (putative passenger) | 6 | 3,6% | 7 | 4,2% |  |  |
|  | Truncating Mutation (putative passenger) | 5 | 3,0% | 4 | 2,4% |  |  |
|  | Inframe Mutation (putative passenger) | 1 | ,6% | 0 | 0,0% |  |  |
|  | No mutation | 153 | 92,7% | 156 | 93,4% |  |  |
| KMT2D | Missense Mutation (putative driver) | 0 | 0,0% | 0 | 0,0% | .152 | .506 |
|  | Truncating Mutation (putative driver) | 2 | 1,2% | 4 | 2,4% |  |  |
|  | Inframe Mutation (putative driver) | 0 | 0,0% | 0 | 0,0% |  |  |
|  | Missense Mutation (putative passenger) | 9 | 5,5% | 3 | 1,8% |  |  |
|  | Truncating Mutation (putative passenger) | 0 | 0,0% | 0 | 0,0% |  |  |
|  | Inframe Mutation (putative passenger) | 0 | 0,0% | 0 | 0,0% |  |  |
|  | No mutation | 154 | 93,3% | 160 | 95,8% |  |  |
| BAP1 | Missense Mutation (putative driver) | 1 | ,6% | 0 | 0,0% | .351 | .585 |
|  | Truncating Mutation (putative driver) | 3 | 1,8% | 8 | 4,8% |  |  |
|  | Inframe Mutation (putative driver) | 0 | 0,0% | 0 | 0,0% |  |  |
|  | Missense Mutation (putative passenger) | 2 | 1,2% | 2 | 1,2% |  |  |
|  | Truncating Mutation (putative passenger) | 0 | 0,0% | 0 | 0,0% |  |  |
|  | Inframe Mutation (putative passenger) | 0 | 0,0% | 0 | 0,0% |  |  |
|  | No mutation | 159 | 96,4% | 157 | 94,0% |  |  |

| Supplementary table 19. Comparative analysis of clinical and molecular features between low AR-Score-7 expression (<=p50) and high AR-Score-7 expression patients. | | | |
| --- | --- | --- | --- |
|  | **Low AR-Score-7**  **(n=168)** | **High AR-Score-7**  **(n=169)** | **p-value** |
| *Age (years)* | 61.1 (SD=12.1) | 58 (SD=13.6) | **.016** |
| *Gender (female:male)* | 44:124 | 63:106 | **.035** |
| *Risk Factors* |  |  | .356 |
| *No risk factors* | 37 (23.3%) | 45 (28.3%) |  |
| *Alcohol consumption* | 57 (35.8%) | 50 (31.4%) |  |
| *Hepatitis B virus* | 33 (20.8%) | 42 (26.4%) |  |
| *Hepatitis C virus* | 19 (11.9%) | 11 (6.9%) |  |
| *NAFLD* | 6 (3.8%) | 3 (1.9%) |  |
| *Other* | 7 (4.4%) | 8 (5%) |  |
| *Child Pugh* |  |  | .279 |
| *A* | 111 (92.5%) | 90 (88.2%) |  |
| *B* | 8 (6.7%) | 12 (11.8%) |  |
| *C* | 1 (0.8%) | - |  |
| *Collection Methods* |  |  | .198 |
| *Single segmentectomy* | 47 (28.3%) | 33 (19.5%) |  |
| *Multiple segmentectomy* | 42 (25.3%) | 36 (21.3%) |  |
| *Lobectomy* | 57 (34.3%) | 72 (42.6%) |  |
| *Lobectomía ampliada* | 10 (6%) | 13 (7.7%) |  |
| *Other* | 10 (6%) | 15 (8.9%) |  |
| *Ishak Score* |  |  | .833 |
| *No fibrosis* | 40 (36%) | 27 (32.5%) |  |
| *Portal fibrosis* | 16 (14.4%) | 13 (15.7%) |  |
| *Fibrous septa* | 12 (10.8%) | 13 (15.7%) |  |
| *Nodular formation and incomplete cirrhosis* | 4 (3.6%) | 4 (4.8%) |  |
| *Established cirrhosis* | 39 (35.1%) | 26 (31.3%) |  |
| *Histologic Grading* |  |  | **.000001** |
| *G1* | 34 (20.2%) | 11 (6.6%) |  |
| *G2* | 92 (54.8%) | 70 (41.9%) |  |
| *G3* | 40 (23.8%) | 76 (45.5%) |  |
| *G4* | 2 (1.2%) | 10 (6%) |  |
| *Pathologic Stage* |  |  | **.005** |
| *Stage I* | 92 (54.8%) | 75 (44.4%) |  |
| *Stage II* | 47 (28%) | 39 (23.1%) |  |
| *Stage IIIa-c* | 29 (17.3%) | 55 (32.5%) |  |
| *Vascular invasion* |  |  | .067 |
| *None* | 103 (68.7%) | 84 (61.3%) |  |
| *Micro* | 43 (28.7%) | 41 (29.9%) |  |
| *Macro* | 4 (2.7%) | 12 (8.8%) |  |
| *Residual Tumour* |  |  | .21 |
| *R0* | 152 (92.7%) | 147 (88.6%) |  |
| *R1* | 3 (1.8%) | 9 (5.4%) |  |
| *R2* | - | - |  |
| *Rx* | 9 (5.5%) | 10 (6%) |  |
| *Mutation Count* | 96.4 (SD=98.3) | 100.9 (SD=120.3) | .79 |
| *Fraction Genome Altered* | 0.2 (SD=0.2) | 0.3 (SD=0.2) | **.0000001** |
| *Aneuploidy Score* | 9.2 (SD=8.1) | 12 (SD=7.6) | **.00005** |
| *AR-CVN* |  |  | - |
| *No* | 164 (100%) | 168 (100%) |  |
| *Amplification* | - | - |  |
| *Deep deletion* | - | - |  |
| *AR-Mutation* |  |  | .609 |
| *Putative driver* | - | - |  |
| *Putative passenger* | 1 (0.6%) | 2 (1.2%) |  |
| *No mutation* | 165 (99.4%) | 166 (98.8%) |  |
| *Progression Free Survival* | 27.2 [18.5 – 35.9] | 18.4 [9.4 – 27.5] | .168 |
| *Overall Survival* | 80.7 [56.0 – 105.5] | 48.9 [25.1 – 72.9] | **.003** |
| ^1^ Mann – Whitney U.  ^2^ Fisher exact test.  ^3^ Chi Square.  ^4^ Log Rank test. | | | |

**Supplementary table 20.** GISTIC analysis of the hepatocellular carcinoma top-10 most common focal amplifications and deletions in cancer-related genes for low and high AR-Score-7 expression groups.

|  | | **AR-Score-7 expression** | | | |  |  |
| --- | --- | --- | --- | --- | --- | --- | --- |
|  |  | **Low** | | **High** | |  |  |
|  |  | *Count* | *%* | *Count* | *%* | *p-value* | *FDR* |
| MUC1 | No | 152 | 92,7% | 146 | 86,9% | .103 | .171 |
|  | Amplification | 12 | 7,3% | 22 | 13,1% |  |  |
|  | Deep Deletion | 0 | 0,0% | 0 | 0,0% |  |  |
| CKS1B | No | 153 | 93,3% | 146 | 86,9% | .066 | .171 |
|  | Amplification | 11 | 6,7% | 22 | 13,1% |  |  |
|  | Deep Deletion | 0 | 0,0% | 0 | 0,0% |  |  |
| LMNA | No | 151 | 92,1% | 146 | 86,9% | .153 | .188 |
|  | Amplification | 13 | 7,9% | 22 | 13,1% |  |  |
|  | Deep Deletion | 0 | 0,0% | 0 | 0,0% |  |  |
| MCL1 | No | 151 | 92,1% | 146 | 86,9% | .153 | .188 |
|  | Amplification | 13 | 7,9% | 22 | 13,1% |  |  |
|  | Deep Deletion | 0 | 0,0% | 0 | 0,0% |  |  |
| RIT1 | No | 152 | 92,7% | 145 | 86,3% | .074 | .171 |
|  | Amplification | 12 | 7,3% | 23 | 13,7% |  |  |
|  | Deep Deletion | 0 | 0,0% | 0 | 0,0% |  |  |
| ARNT | No | 152 | 92,7% | 145 | 86,3% | .074 | .171 |
|  | Amplification | 12 | 7,3% | 23 | 13,7% |  |  |
|  | Deep Deletion | 0 | 0,0% | 0 | 0,0% |  |  |
| EXT1 | No | 147 | 89,6% | 145 | 86,3% | .329 | .329 |
|  | Amplification | 16 | 9,8% | 23 | 13,7% |  |  |
|  | Deep Deletion | 1 | ,6% | 0 | 0,0% |  |  |
| MYC | No | 148 | 90,2% | 143 | 85,1% | .17 | .188 |
|  | Amplification | 15 | 9,1% | 25 | 14,9% |  |  |
|  | Deep Deletion | 1 | ,6% | 0 | 0,0% |  |  |
| YY1AP1 | No | 152 | 92,7% | 146 | 86,9% | .103 | .171 |
|  | Amplification | 12 | 7,3% | 22 | 13,1% |  |  |
|  | Deep Deletion | 0 | 0,0% | 0 | 0,0% |  |  |
| TPM3 | No | 153 | 93,3% | 145 | 86,3% | **.046** | .171 |
|  | Amplification | 11 | 6,7% | 23 | 13,7% |  |  |
|  | Deep Deletion | 0 | 0,0% | 0 | 0,0% |  |  |

**Supplementary table 21.** Mutational signature of the hepatocellular carcinoma top-10 most common cancer-related mutated genes for low and high AR-Score-7 expression groups.

|  | | **AR-Score-7 expression** | | | |  |  |
| --- | --- | --- | --- | --- | --- | --- | --- |
|  |  | **Low** | | **High** | |  |  |
|  |  | *Count* | *%* | *Count* | *%* | *p-value* | *FDR* |
| TP53 | Missense Mutation (putative driver) | 17 | 10,4% | 42 | 25,0% | **.003** | **.03** |
|  | Truncating Mutation (putative driver) | 17 | 10,4% | 22 | 13,1% |  |  |
|  | Inframe Mutation (putative driver) | 2 | 1,2% | 1 | ,6% |  |  |
|  | Missense Mutation (putative passenger) | 0 | 0,0% | 0 | 0,0% |  |  |
|  | Truncating Mutation (putative passenger) | 0 | 0,0% | 0 | 0,0% |  |  |
|  | Inframe Mutation (putative passenger) | 0 | 0,0% | 0 | 0,0% |  |  |
|  | No mutation | 128 | 78,0% | 103 | 61,3% |  |  |
| CTNNB1 | Missense Mutation (putative driver) | 55 | 33,5% | 30 | 17,9% | **.008** | **0.04** |
|  | Truncating Mutation (putative driver) | 0 | 0,0% | 0 | 0,0% |  |  |
|  | Inframe Mutation (putative driver) | 1 | ,6% | 0 | 0,0% |  |  |
|  | Missense Mutation (putative passenger) | 1 | ,6% | 3 | 1,8% |  |  |
|  | Truncating Mutation (putative passenger) | 1 | ,6% | 0 | 0,0% |  |  |
|  | Inframe Mutation (putative passenger) | 0 | 0,0% | 0 | 0,0% |  |  |
|  | No mutation | 106 | 64,6% | 135 | 80,4% |  |  |
| ALB | Missense Mutation (putative driver) | 0 | 0,0% | 0 | 0,0% | **.034** | 0.113 |
|  | Truncating Mutation (putative driver) | 0 | 0,0% | 0 | 0,0% |  |  |
|  | Inframe Mutation (putative driver) | 0 | 0,0% | 0 | 0,0% |  |  |
|  | Missense Mutation (putative passenger) | 5 | 3,0% | 7 | 4,2% |  |  |
|  | Truncating Mutation (putative passenger) | 19 | 11,6% | 8 | 4,8% |  |  |
|  | Inframe Mutation (putative passenger) | 3 | 1,8% | 0 | 0,0% |  |  |
|  | No mutation | 137 | 83,5% | 153 | 91,1% |  |  |
| PCLO | Missense Mutation (putative driver) | 0 | 0,0% | 0 | 0,0% | .691 | 0.691 |
|  | Truncating Mutation (putative driver) | 0 | 0,0% | 0 | 0,0% |  |  |
|  | Inframe Mutation (putative driver) | 0 | 0,0% | 0 | 0,0% |  |  |
|  | Missense Mutation (putative passenger) | 15 | 9,1% | 15 | 8,9% |  |  |
|  | Truncating Mutation (putative passenger) | 4 | 2,4% | 2 | 1,2% |  |  |
|  | Inframe Mutation (putative passenger) | 0 | 0,0% | 0 | 0,0% |  |  |
|  | No mutation | 145 | 88,4% | 151 | 89,9% |  |  |
| LRP1B | Missense Mutation (putative driver) | 0 | 0,0% | 0 | 0,0% | .147 | 0.245 |
|  | Truncating Mutation (putative driver) | 0 | 0,0% | 0 | 0,0% |  |  |
|  | Inframe Mutation (putative driver) | 0 | 0,0% | 0 | 0,0% |  |  |
|  | Missense Mutation (putative passenger) | 9 | 5,5% | 16 | 9,5% |  |  |
|  | Truncating Mutation (putative passenger) | 1 | ,6% | 4 | 2,4% |  |  |
|  | Inframe Mutation (putative passenger) | 0 | 0,0% | 0 | 0,0% |  |  |
|  | No mutation | 154 | 93,9% | 148 | 88,1% |  |  |
| ARID1A | Missense Mutation (putative driver) | 1 | ,6% | 0 | 0,0% | .491 | 0.545 |
|  | Truncating Mutation (putative driver) | 7 | 4,3% | 12 | 7,1% |  |  |
|  | Inframe Mutation (putative driver) | 0 | 0,0% | 0 | 0,0% |  |  |
|  | Missense Mutation (putative passenger) | 3 | 1,8% | 4 | 2,4% |  |  |
|  | Truncating Mutation (putative passenger) | 0 | 0,0% | 0 | 0,0% |  |  |
|  | Inframe Mutation (putative passenger) | 0 | 0,0% | 0 | 0,0% |  |  |
|  | No mutation | 153 | 93,3% | 152 | 90,5% |  |  |
| AXIN1 | Missense Mutation (putative driver) | 0 | 0,0% | 0 | 0,0% | .064 | 0.16 |
|  | Truncating Mutation (putative driver) | 7 | 4,3% | 16 | 9,5% |  |  |
|  | Inframe Mutation (putative driver) | 0 | 0,0% | 0 | 0,0% |  |  |
|  | Missense Mutation (putative passenger) | 2 | 1,2% | 0 | 0,0% |  |  |
|  | Truncating Mutation (putative passenger) | 0 | 0,0% | 0 | 0,0% |  |  |
|  | Inframe Mutation (putative passenger) | 0 | 0,0% | 0 | 0,0% |  |  |
|  | No mutation | 155 | 94,5% | 152 | 90,5% |  |  |
| PRKDC | Missense Mutation (putative driver) | 0 | 0,0% | 0 | 0,0% | .386 | 0.525 |
|  | Truncating Mutation (putative driver) | 0 | 0,0% | 0 | 0,0% |  |  |
|  | Inframe Mutation (putative driver) | 0 | 0,0% | 0 | 0,0% |  |  |
|  | Missense Mutation (putative passenger) | 8 | 4,9% | 5 | 3,0% |  |  |
|  | Truncating Mutation (putative passenger) | 6 | 3,7% | 3 | 1,8% |  |  |
|  | Inframe Mutation (putative passenger) | 1 | ,6% | 0 | 0,0% |  |  |
|  | No mutation | 149 | 90,9% | 160 | 95,2% |  |  |
| KMT2D | Missense Mutation (putative driver) | 0 | 0,0% | 0 | 0,0% | .13 | 0.245 |
|  | Truncating Mutation (putative driver) | 4 | 2,4% | 2 | 1,2% |  |  |
|  | Inframe Mutation (putative driver) | 0 | 0,0% | 0 | 0,0% |  |  |
|  | Missense Mutation (putative passenger) | 9 | 5,5% | 3 | 1,8% |  |  |
|  | Truncating Mutation (putative passenger) | 0 | 0,0% | 0 | 0,0% |  |  |
|  | Inframe Mutation (putative passenger) | 0 | 0,0% | 0 | 0,0% |  |  |
|  | No mutation | 151 | 92,1% | 163 | 97,0% |  |  |
| BAP1 | Missense Mutation (putative driver) | 1 | ,6% | 0 | 0,0% | .42 | 0.525 |
|  | Truncating Mutation (putative driver) | 4 | 2,4% | 7 | 4,2% |  |  |
|  | Inframe Mutation (putative driver) | 0 | 0,0% | 0 | 0,0% |  |  |
|  | Missense Mutation (putative passenger) | 3 | 1,8% | 1 | ,6% |  |  |
|  | Truncating Mutation (putative passenger) | 0 | 0,0% | 0 | 0,0% |  |  |
|  | Inframe Mutation (putative passenger) | 0 | 0,0% | 0 | 0,0% |  |  |
|  | No mutation | 156 | 95,1% | 160 | 95,2% |  |  |

**Differential expression of ARGs in normal liver tissue vs. HCC tissue.**

In the TCGA hepatocellular cancer dataset only 50 subjects presented the RNAseq data of both, normal and tumoral tissue. We extracted all the data and calculated the different AR-Scores as described in the methods section. Non-parametric test (Wilcoxon signed rank test) was used to test the differences between normal and tumoral probes. Higher AR-Score-21 and AR-Score-13 means were identified in normal than tumoral probes (AR-Score-21: p<0.05; AR-Score-13: p=0.023). However, no significant differences were identified between normal and tumoral probes for AR-Score-6 (p=0.658) and AR-Score-7 (p=0.463). It should be highlighted that only 15 patients with histological grade 3 and no patient with histological grade 4 were included in this secondary analysis. Thus, high-grade hepatocellular carcinomas are underrepresented. The differential expression analysis between normal and tumoral probes showed differences in some ARGs. On one hand, normal probes presented higher expression of ELL2, **GNMT**, **HERC3**, KLK3, KLF4, **MAF**, **NFKBIA**, NKX3, NNMT, PTGER4, SGK1, **SLC22A3**, **TMPRSS2** and ZBTB10 (supplementary table 22). Genes in bold type were significantly downregulated in high histological grade HCC in the whole cohort of tumoral probes in the present study (see results section). On the other hand, tumoral probes presented higher expression of **ABCC4**, ATP1A1, CENPN, **NDRG1**, PYGB, RELL2, **SLC26A2** and **SSR3** (supplementary table 22). Genes in bold type were significantly upregulated in the whole cohort of tumoral probes in the present study (see results section).

| **Supplementary table 22. Differential ARGs expression in normal liver tissue and HCC tissue (50 patients with paired probes). Red rows represent genes upregulated in tumoral probes, while green rows represent ARGs upregulated in normal liver tissue probes.** | | | | | |
| --- | --- | --- | --- | --- | --- |
|  | **Normal Tissue** | | **Tumoral Tissue** | |  |
| **GENE** | **Mean Exp.** | **SD** | **Mean Exp.** | **SD** | **p-value** |
| ABCC4 | -0.23 | 0.44 | 0.23 | 1.31 | 0.011 |
| ACSL3 | -0.15 | 0.73 | 0.15 | 1.20 | 0.092 |
| ATP1A1 | -0.32 | 0.51 | 0.32 | 1.24 | 0.001 |
| C1orf116 | 0.32 | 0.45 | -0.32 | 1.27 | 0.004 |
| CENPN | -0.25 | 0.58 | 0.25 | 1.25 | 0.027 |
| EAF2 | -0.08 | 0.93 | 0.08 | 1.07 | 0.582 |
| ELL2 | 0.43 | 0.74 | -0.43 | 1.04 | 0.000 |
| FKBP5 | -0.03 | 0.93 | 0.03 | 1.07 | 0.605 |
| GNMT | 0.54 | 0.44 | -0.54 | 1.11 | 0.000 |
| HERC3 | 0.47 | 0.48 | -0.47 | 1.16 | 0.000 |
| HK3 | 0.64 | 0.54 | -0.64 | 0.94 | 0.000 |
| KLF4 | 0.55 | 0.64 | -0.55 | 1.00 | 0.000 |
| KLK2 | -0.08 | 1.10 | 0.01 | 1.04 | - |
| MAF | 0.58 | 0.78 | -0.59 | 0.84 | 0.000 |
| MCEE | 0.56 | 0.47 | -0.55 | 1.08 | 0.082 |
| MED28 | 0.17 | 0.91 | -0.17 | 1.06 | 0.082 |
| MPHOSPH9 | -0.07 | 0.75 | 0.07 | 1.20 | 0.199 |
| NDRG1 | -0.22 | 0.55 | 0.23 | 1.27 | 0.023 |
| NELL2 | -0.13 | 0.88 | 0.13 | 1.11 | 0.140 |
| NFKBIA | 0.42 | 0.77 | -0.42 | 1.03 | 0.000 |
| NKX3-1 | 0.62 | 0.39 | -0.62 | 1.04 | 0.000 |
| NNMT | 0.67 | 0.41 | -0.67 | 0.97 | 0.000 |
| PMEPA1 | -0.03 | 0.63 | 0.03 | 1.27 | 0.806 |
| PTGER4 | 0.40 | 0.70 | -0.40 | 1.10 | 0.000 |
| PYGB | -0.67 | 0.44 | 0.67 | 0.96 | 0.000 |
| RELL2 | -0.50 | 0.62 | 0.49 | 1.06 | 0.000 |
| SGK1 | 0.61 | 0.68 | -0.61 | 0.89 | 0.000 |
| SLC22A3 | 0.56 | 0.24 | -0.56 | 1.15 | 0.000 |
| SLC26A2 | -0.64 | 0.56 | 0.64 | 0.93 | 0.000 |
| SSR3 | -0.58 | 0.59 | 0.58 | 1.00 | 0.000 |
| TMPRSS2 | 0.44 | 0.54 | -0.44 | 1.16 | 0.000 |
| VEGFA | 0.00 | 0.00 | 0.06 | 1.22 | 0.988 |
| ZBTB10 | 0.40 | 0.67 | -0.48 | 1.08 | 0.000 |
